# Amplification Dynamics of Platy-1 Retrotransposons in the Cebidae Platyrrhine Lineage

**DOI:** 10.1093/gbe/evz062

**Published:** 2019-03-19

**Authors:** Jessica M Storer, Jackson R Mierl, Sarah A Brantley, Breanna Threeton, Yahor Sukharutski, Lydia C Rewerts, Corey P St. Romain, Madeline M Foreman, Jasmine N Baker, Jerilyn A Walker, Joseph D Orkin, Amanda D Melin, Kimberley A Phillips, Miriam K Konkel, Mark A Batzer

**Affiliations:** 1 Department of Biological Sciences, Louisiana State University; 2 Department of Anthropology and Archaeology & Department of Medical Genetics, University of Calgary, Alberta, Canada; 3 Alberta Children’s Hospital Research Institute, N.W. Calgary, Alberta, Canada; 4 Department of Psychology, Trinity University; 5 Southwest National Primate Research Center, Texas Biomedical Research Institute, San Antonio, Texas; 6 Department of Genetics & Biochemistry, Clemson University

**Keywords:** insertion, polymorphism, evolution, subfamilies

## Abstract

Platy-1 elements are Platyrrhine-specific, short interspersed elements originally discovered in the *Callithrix jacchus* (common marmoset) genome. To date, only the marmoset genome has been analyzed for Platy-1 repeat content. Here, we report full-length Platy-1 insertions in other New World monkey (NWM) genomes (*Saimiri boliviensis*, squirrel monkey; *Cebus imitator*, capuchin monkey; and *Aotus nancymaae*, owl monkey) and analyze the amplification dynamics of lineage-specific Platy-1 insertions. A relatively small number of full-length and lineage-specific Platy-1 elements were found in the squirrel, capuchin, and owl monkey genomes compared with the marmoset genome. In addition, only a few older Platy-1 subfamilies were recovered in this study, with no Platy-1 subfamilies younger than Platy-1-6. By contrast, 62 Platy-1 subfamilies were discovered in the marmoset genome. All of the lineage-specific insertions found in the squirrel and capuchin monkeys were fixed present. However, ∼15% of the lineage-specific Platy-1 loci in *Aotus* were polymorphic for insertion presence/absence. In addition, two new Platy-1 subfamilies were identified in the owl monkey genome with low nucleotide divergences compared with their respective consensus sequences, suggesting minimal ongoing retrotransposition in the *Aotus* genus and no current activity in the *Saimiri*, *Cebus*, and *Sapajus* genera. These comparative analyses highlight the finding that the high number of Platy-1 elements discovered in the marmoset genome is an exception among NWM analyzed thus far, rather than the rule. Future studies are needed to expand upon our knowledge of Platy-1 amplification in other NWM genomes.

## Introduction

Transposable elements (TEs) are discrete pieces of DNA that are able to move from one genomic location to another. These elements can be broadly categorized based on their movement mechanism, either “cut-and-paste” or “copy-and-paste.” The former category includes DNA transposable elements that mobilize via a DNA intermediate. (Hellen and Brookfield 2013). The latter category includes retrotransposable elements that move via an RNA intermediate (Batzer and Deininger 2002; Konkel et al. 2010). In primates, retrotransposable nonautonomous short interspersed elements (SINEs) such as *Alu* elements (Houck et al. 1979) and autonomous long interspersed elements (LINEs) called L1s make up roughly 10% and 17% of the genome by mass, respectively (Lander et al. 2001; Batzer and Deininger 2002; Richardson et al. 2015). The high copy number *Alu* elements are ∼300 base pairs (bp) long with a dimeric structure, separated by a middle A-rich region. These elements are derived from the 7SL RNA, a component of the signal recognition particle (Batzer and Deininger 2002; Konkel et al. 2010). These elements are mobilized via a retrotransposition mechanism called target-primed reverse transcription (TPRT) (Luan et al. 1993; Morrish et al. 2002). However, *Alu* does not code for the proteins required for TPRT, and must rely on the protein products of L1s for movement (Dewannieux et al. 2003). Hallmarks of TPRT include a 5′ and 3′ flanking target site duplication (TSD), endonuclease cleavage site and a 3′ A-rich tail, allowing for additional elements that mobilize via this mechanism to be identified (Morrish et al. 2002). The manner of *Alu* mobilization generates random and nonrandom mutations. The nonrandom mutations are termed diagnostic mutations and serve to divide *Alu* repeats into subfamilies (Jurka and Smith 1988; Deininger et al. 1992). The independent amplification of *Alu* repeats that occurs in separate lineages may lead to the propagation of new mobile element subfamilies.

New World monkeys (NWM) are a diverse group of primates belonging to the parvorder Platyrrhini. These small to midsized primates are located in Central and South America and belong to one of three families: Cebidae (small, arboreal monkeys), Atelidae (large monkeys with prehensile tails), or Pitheciidae (herbivorous monkeys) (Schneider and Sampaio 2015). Since the first study of NWM cladistics, the phylogeny of NWM has been under debate (Ray and Batzer 2005; Ray et al. 2005; Osterholz et al. 2009). This is in part due to poor fossil records (Perez et al. 2013) making divergence times and speciation events difficult to pinpoint. In addition, different morphological and molecular markers have produced conflicting results for some portions of the NWM tree (Dumas et al. 2007; Pacheco et al. 2010; de Oliveira et al. 2012; Hiroshige et al. 2015; Capozzi et al. 2016). However, there is general agreement of the three NWM families as well as the genera included. Within the Cebidae family, there are three clades whose phylogenetic relationship is still being studied: *Aotinae, Cebinae*, and *Callitrichinae* (Schneider and Sampaio 2015). *Aotinae* includes night monkeys, belonging to the *Aotus* genus, *Cebinae* includes the extant genus *Saimiri* and capuchin monkeys which include two extant genera, *Cebus* and *Sapajus* (formerly subsumed into one genus, *Cebus*) (Alfaro et al. 2012), and *Callitrichinae* which includes marmosets (genera *Callithrix, Callimico, Cebuella*, and *Mico)* and tamarins (genera *Leontopithecus* and *Saguinus*) (Garbino and Martins-Junior 2018)*.* While reported divergence times and radiation of these three clades have varied, there is a general consensus that the rapid radiation occurred over a short time of 1–2 Myr. Estimates of when this divergence occurred range from 19.25 Ma (Perelman et al. 2011) to 23.2 Ma (Schneider 2000).

Recently, a new retrotransposable element was discovered in the common marmoset genome and subsequently found to be specific to the Platyrrhini parvorder. Deemed, “Platy-1,” these ∼100 bp elements have the hallmarks of movement via TPRT (Konkel et al. 2016). In addition, these elements share some structure and sequence similarity with *Alu* elements, a primate-specific SINE, suggesting that Platy-1 likely originated from an *Alu* element and is 7SL RNA derived (Konkel et al. 2016). Roughly 2,200 Platy-1 elements were found in the common marmoset genome [calJac3], prompting a closer look at other NWMs with whole genome sequence data available.

Although once thought to be “junk” DNA (Kazazian 2011), TEs have had an unexpected influence on primate biology in terms of disease, phenotypes, and evolution. TEs can cause genomic instability via double-stranded breaks (Belgnaoui et al. 2006; Gasior et al. 2006) and nonhomologous recombination (Han et al. 2005a; Startek et al. 2015), potentially influencing meiosis, depending upon the location of the insertion as well as resulting in the contraction or expansion of genome size. In addition, based on their insertional location, TEs can affect transcriptional control via influencing alternative splicing if inserted into the coding region of a gene, disrupting the formation of a gene product, or influencing the methylation status of the TE’s surrounding environment (Cordaux and Batzer 2009). It is therefore informative to understand the amplification dynamics of mobile elements in order to understand how genomes have evolved, particularly because of parallel evolution in which many mobile elements may be active in multiple lineages simultaneously. Due to parallel evolution, each NWM lineage will have its own unique distribution of not only TE families but also distinctive subfamilies within each family. For example, the discovery of 46 *Saimiri* lineage-specific *Alu* subfamilies was recently reported (Baker et al. 2017), most of which derived from the larger group of established *Alu*Ta subfamilies. *Alu*Ta subfamilies are specific to NWM as the result of a unique fusion event between two anthropoid *Alu*S subfamilies (Ray and Batzer 2005) and have been used to study phylogenetic relationships.

The purpose of this study was to characterize the Platy-1 elements found in the current genome assemblies of other Platyrrhine primates in order to determine the quantity of Platy-1 elements as well as the amplification dynamics in comparison to the common marmoset genome (Konkel et al. 2016).

## Materials and Methods

### Platy-1 Lineage Specificity

**Fig. 1.**
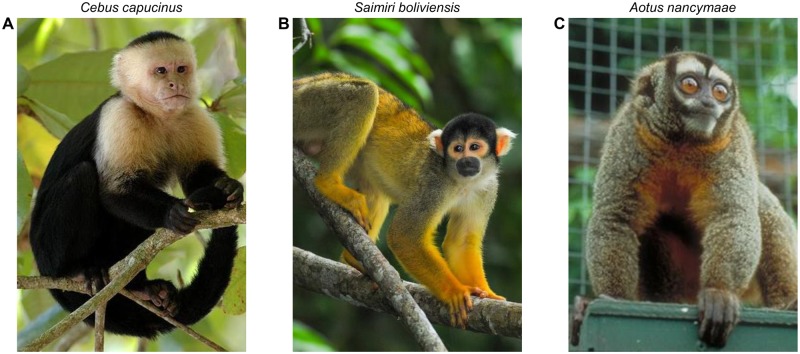
—Photographs representative of the NWM used in this study. (*A*) *Cebus capucinus* or capuchin monkey, (*B*) *Saimiri boliviensis* or squirrel monkey, and (*C*) *Aotus nancymaae* or owl monkey (Cawthon Lang 2005).

The capuchin monkey (*Cebus imitator*), owl monkey (*Aotus nancymaae*), and squirrel monkey (*Saimiri boliviensis*) genomes were obtained from NCBI (cebus-Cebus_imitator-1.0; owl-Anan_1.0) and the University of California Santa Cruz (UCSC) genome browser (squirrel-saiBol1). Assembly statistics for each genome are available in table 1 and representative photographs for each species are shown in fig. 1). These genomes were analyzed for Platy-1 elements using RepeatMasker (RepeatMasker-Open-4.0) utilizing a custom library of the 62 Platy-1 subfamilies previously defined (Konkel et al. 2016) and all current *Alu* subfamily consensus sequences obtained from RepBase (Jurka et al. 2005). Full-length Platy-1 elements were defined as possessing a start position no less than 4 bp and an end position not shorter than two nucleotides prior to the A-tail within the consensus sequence (Konkel et al. 2016). Full-length elements were extracted from the RepeatMasker output using a custom python script. These elements, along with 600 bp of flanking sequence on both the 5′ and the 3′ ends of the Platy-1 insertion, were compared with the common marmoset (*Callithrix jacchus*/calJac3) and the remaining NWM genomes using a locally installed version of BLAT (Kent 2002) to determine lineage specificity. Specificity was determined by visualizing the BLAT alignments using pslPretty and observing a ∼100 bp gap. For each locus an alignment file was generated in BioEdit (Hall 1999) to be used for the design of oligonucleotide primers.

**Table 1:** Genome Assembly Statistics

Genome	Common Name	Assembly	N50 (Contig)	N50 (Scaffold)	Coverage	Number of Gaps	Size (bp)
*Cebus imitator*	Capuchin monkey	Cebus_imitator-1.0	41,196	5,274,112	81×	133,441	2.72×10^9^
*Saimiri boliviensis*	Squirrel monkey	saiBol1	38,823	18,744,880	80×	148,728	2.61×10^9^
*Aotus nancymaae*	Owl monkey	Anan_1.0	28,503	8,280,397	113.4×	215,259	2.93×10^9^

Note.—The assembly statistics for the NWM genomes used in this study are shown above.

### Platy-1 Shared Elements

To analyze shared elements among NWM, the whole-genome aligner mugsy (Angiuoli and Salzberg 2011) was utilized. All Platy-1 elements with flanking sequence for each lineage (squirrel, capuchin, and owl monkeys as well as marmoset) were put into one FASTA file. The resulting four FASTA files were then aligned as if they were whole genomes using the whole genome function in mugsy. The output .maf file was visualized using GMAJ (globin.bx.psu.edu/dist/gmaj/; last accessed March 26, 2019) and manually assessed for alignment precision. This analysis proved fruitful for obtaining elements that were shared among all four of the genomes analyzed. However, elements that were computationally predicted to be shared between only two or three of the four genomes, typically had gaps in the sequence assembly of the genome(s) in which the insertion was absent, thus obscuring any potential phylogenetically informative data. To overcome this limitation, for the pool of elements not shared among all four genomes, we used BLAT followed by a custom python script to obtain orthologous sequences from each genome and then aligned all four sequences for each locus using BioEdit (Hall 1999).

### Oligonucleotide Primer Design

The loci determined to contain Platy-1 elements unique to each NWM were put into individual files containing the orthologous sequences from marmoset, squirrel monkey, owl monkey, and capuchin monkey genomes. These sequences were aligned using CLUSTALW (Thompson et al. 1994) and/or MUSCLE (Edgar 2004). Forward and reverse oligonucleotide primers for polymerase chain reaction (PCR) were designed using Primer3 (v.0.4.0) and checked in BioEdit to ensure minimal mismatches to allow for the amplification of a PCR product in all genomes specified. In silico PCR was used to confirm the oligonucleotide primers would amplify only one product in multiple species. The same process was followed for the shared Platy-1 elements (supplementary file 1, Supplementary Material online).

### DNA Samples

DNA samples are described in supplementary file 2, Supplementary Material online. Briefly, there were four panels utilized for this study: a NWM panel, a squirrel monkey panel, an owl monkey panel, and a capuchin monkey panel. The NWM panel contained three Old World monkeys (OWM) and sixteen NWM species representing the three NWM families. This DNA panel was used to screen elements for lineage-specificity. The squirrel monkey panel included DNA samples from 32 individuals of the genus *Saimiri* representing five species, the owl monkey panel included DNA samples from 23 individuals of the genus *Aotus* representing five species, and the capuchin monkey panel included DNA from 14 different capuchin monkeys, 8 *Cebus apella*, now considered genus *Sapajus apella* (Alfaro et al. 2012), and 6 individuals from genus *Cebus* including the *Cebus imitator* sample used as the reference genome.

### Polymerase Chain Reaction Amplification

PCR amplification was performed in 25 µl reactions containing 25 µg of template DNA, 200 nM of each primer, 1.5 mM MgCl_2_, 10× PCR buffer (1×: 50 mM KCl; 10 mM Tris–HCl, pH 8.4), 0.2 mM dNTPs, and 1 unit of *Taq* DNA polymerase. The PCR reaction protocol is as follows: 94°C for 1 min, 32 cycles of denaturation at 94°C for 30 s, 30 s at the appropriate annealing temperature (typically 57°C), extension at 72°C for 30 s, followed by a final 72°C extension step for 2 min. Gel electrophoresis was performed on a 2% agarose gel containing 0.2 µg/mL ethidium bromide for 60 min at 180 V. UV fluorescence was used to visualize the DNA fragments using a BioRad ChemiDoc XRS imaging system (Hercules, CA). If PCR results were weak or unresolved, the PCR reaction was repeated using hot-start with the JumpStart *Taq* DNA polymerase kit (Sigma Aldrich). Genotypes were recorded in a Microsoft Excel worksheet as (0, 0) homozygous absent, (1, 1) homozygous present, or (1, 0) for heterozygous (supplementary file 3, Supplementary Material online).

### Age of Platy-1 Elements

The age of the Platy-1 elements was estimated by utilizing the percent divergence of each element to the subfamily consensus sequence, a feature available in the RepeatMasker output. The mutation rate of 0.006024 per base per million years (my) (Konkel et al. 2016) was used to estimate the age of the Platy-1 subfamilies. This rate is the composite of the substitution rate of the crown Platyrrhines and the crown Cebidae (Perez et al. 2013; Konkel et al. 2016). This mutation rate, along with the equation:

T=D/t

where *D* is the percent divergence and *t* is the substitution rate, was used to calculate the age (T) (my) of the Platy-1 elements (supplementary file 4, Supplementary Material online).

## Results

### Lineage-Specific Platy-1 Insertions in NWM

**Fig. 2.**
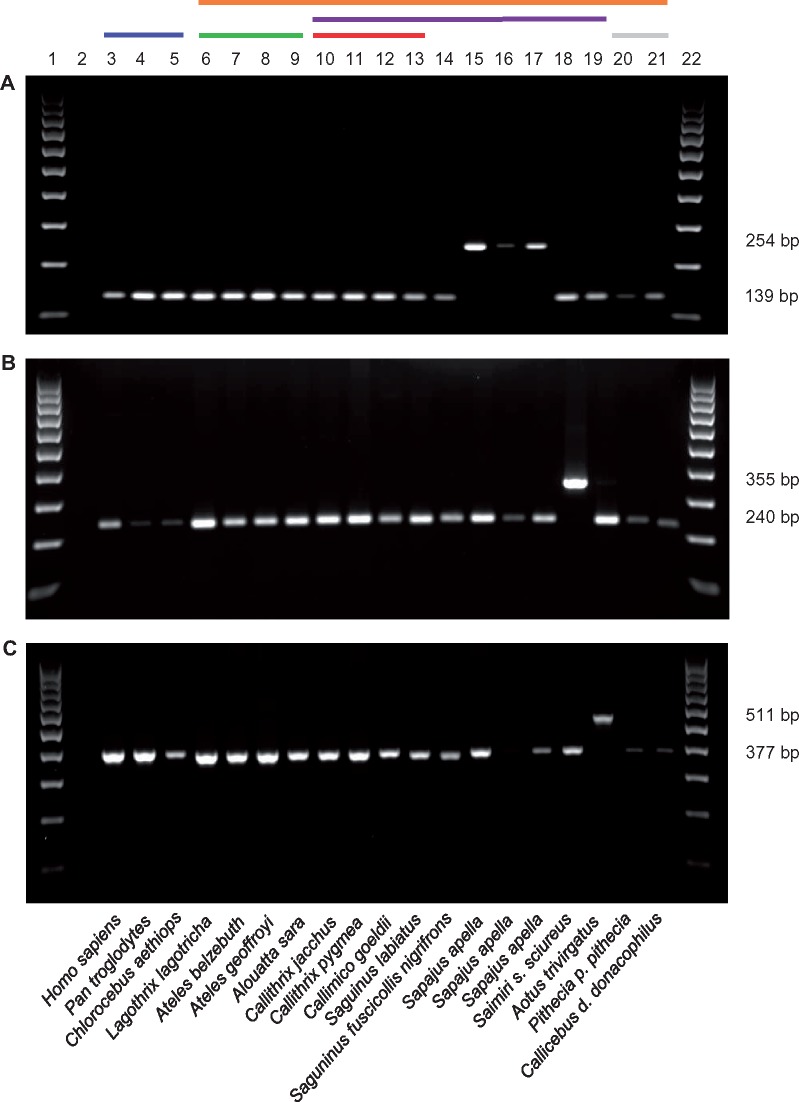
—Lineage-specific Platy-1 elements. (*A*) The presence of the Ceb_5 capuchin monkey specific Platy-1 element is indicated by the higher of the two bands present (254-bp band), while the absence is indicated by the lower of the two bands present (139-bp band). (*B*) The presence of the Ply4a-27 squirrel monkey specific Platy-1 element is indicated by the higher of the two bands present (355-bp band), while the absence is indicated by the lower of the two bands present (240-bp band). (*C*) The presence of the U_OM_89423_v3 owl monkey specific Platy-1 element is indicated by the higher of the two bands present (511-bp band), while the absence is indicated by the lower of the two bands present (377-bp band). Lanes: 1: 100-bp ladder; 2: TLE (negative control); 3: Human (HeLa); 4: Chimpanzee; 5: African green monkey; 6: Wooly monkey; 7: White-bellied spider monkey; 8: Black-handed howler monkey; 9: Bolivian red howler monkey; 10: Common marmoset; 11: Pygmy marmoset; 12: Goeldi’s marmoset; 13: Red-chested mustached tamarin; 14: Geoffroys saddle-back tamarin; 15–17: Capuchin monkey; 18: Squirrel monkey; 19: Owl monkey; 20: Northern white-faced saki; 21: Bolivian gray titi; 22: 100-bp ladder. The bars above the gel electrophoresis image indicate the following: Blue-Old World Monkey; Gold-NWM; Green-Atelidae; Purple-Cebidae; Red-Callithrichinae; Gray-Pitheciidae. Scientific names of the primates are indicated below the gel images.

**Fig. 3.**
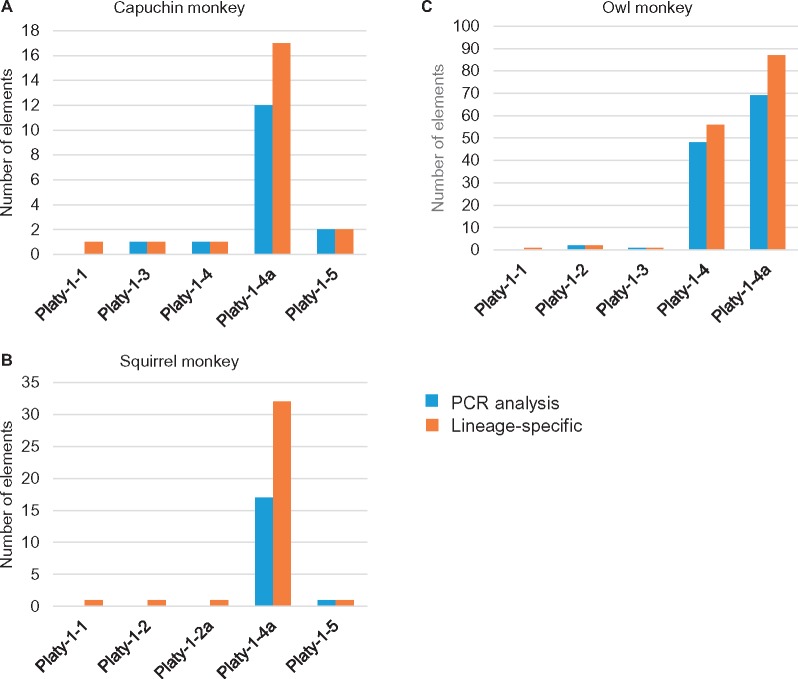
—Predicted lineage-specific Platy-1 elements and PCR analyses. A comparison of the number of the predicted lineage-specific and PCR-analyzed loci is shown, as well as the number of elements belonging to each Platy-1 subfamily in the aforementioned categories. (*A*) Capuchin monkey, (*B*) squirrel monkey, and (*C*) owl monkey. Note the differences in scale between capuchin and squirrel versus owl monkeys.

A total of 387, 605 and 335 Platy-1 loci were retrieved from the RepeatMasker analysis of the capuchin [Cebus_imitator-1.0], owl monkey [Anan_1.0], and squirrel monkey [saiBol1] genomes, respectively (table 2). Of these, 171, 378, and 158 were determined to be full-length insertions, as previously defined (see Materials and Methods; Konkel et al. 2016). These values are strikingly low as compared with the 2,268 full-length Platy-1 elements previously identified in marmoset [calJac3] (Konkel et al. 2016). In the capuchin genome, there were 22 predicted lineage-specific Platy-1 insertions, with 16 insertions conducive to locus-specific PCR (table 2, figs. 2*A* and 3*A*). The squirrel monkey genome had a similarly low number of lineage-specific insertions, 36, with 18 of these analyzed by PCR (table 2, figs. 2*B* and 3*B*). With 145 loci, the owl monkey genome had the largest number of lineage-specific insertions of the three NWM genomes investigated. Of these, 119 insertions were analyzed using locus-specific PCR (table 2, figs. 2*C* and 3*C*).

**Table 2:** Platy-1 Element Summary

	Total	Full-Length	Lineage-Specific	PCR
Capuchin monkey	387	171	22	16
Squirrel monkey	335	158	36	18
Owl monkey	605	378	145	119

Note.—The table shows the total Platy-1 elements extracted from the RepeatMasker output (Total), the full-length elements extracted from the RepeatMasker output (Full-length), the elements that were predicted to be lineage-specific, and full-length loci analyzed using locus specific PCR for each NWM.

**Fig. 4.**
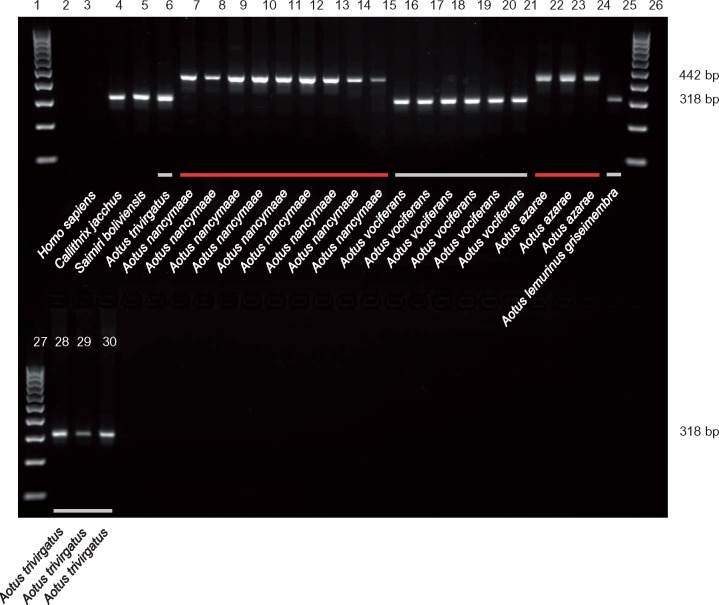
—Polymorphic Platy-1 element in Owl monkeys. A gel image was generated subsequent to a PCR for site U_OM_87201 using primers that utilized the DNA from the owl monkey DNA panel. A band indicating a filled site is 442 bp while an empty site is indicated by a 318-bp band. Lanes: 1: 100-bp ladder; 2: TLE (negative control); 3: Human (HeLa); 4: Common marmoset; 5: Bolivian squirrel monkey; 6: Three-striped owl monkey; 7–15: Nancy Ma’s night monkey; 16–21: Noisy owl monkey (Spix’s night monkey); 22–24: Azara’s night monkey; 25: Panamanian night monkey; 26–27: 100-bp ladder; 28–30: Three-striped owl monkey. Scientific names of the primates are indicated below the gel images. The bars beneath the gel electrophoresis bands indicate the following: red–red-necked owl monkeys and gray–gray-necked owl monkeys. Note that all of the filled sites on the electrophoresis gel image belong to DNA samples corresponding to red-necked owl monkeys, while empty sites correspond to gray-necked owl monkeys.

Of the capuchin monkey lineage-specific Platy-1 insertions, the majority belonged to the 4a subfamily (fig. 3*A*). All of the 16 loci subjected to PCR (see Materials and Methods) were homozygous for the presence of the insertion (supplementary file 3, Supplementary Material online). A similar trend was observed for the squirrel monkey lineage-specific insertions, as all 18 PCR-analyzed loci in this lineage were fixed present and the majority of these insertions also belonged to the 4a subfamily (supplementary file 3, Supplementary Material online and fig. 3*B*). The owl monkey genome had a considerably higher number of lineage-specific insertions, with the majority of the elements being either 4 or 4a subfamily members (table 2 andfig. 3*C*). Of the 119 loci analyzed by PCR, 88 were homozygous present, while 31 remained polymorphic for insertion presence/absence among 23 *Aotus* individuals analyzed (supplementary file 3, Supplementary Material online; figs. 2*C* and 4). The *Aotus* genus was the only one of four genera in this study to show evidence of ongoing Platy-1 mobilization. Given the rapid radiation of these four genera as discussed in a review article by Schneider and Sampaio (2015), our data suggests the emergence of the 4a Platy-1 subfamily approximately between 19 and 20 Ma.

Among the 31 polymorphic loci identified in the owl monkey genome, the allele frequency variation across 23 *Aotus* individuals revealed a distinct separation between the two recognized groups of owl monkey, red- and gray-necked (Menezes et al. 2010), for at least three loci. For these loci there was a clear separation of species with (homozygous present) and without (homozygous absent) a Platy-1 insertion (supplementary file 3, Supplementary Material online and fig. 4), reflecting the red-necked (*A. nancymaae, A. azarae*) and gray-necked (*A. lemurinus, A. trivirgatus, A. vociferans*) divergence seen in South America (Menezes et al. 2010). The majority of the lineage-specific Platy-1 elements discovered in this study were members of previously-defined Platy-1 subfamilies 4 or 4a based on the subfamily consensus sequences reported in Konkel et al. (2016). Evidence of recent mobilization within *Aotus*, and no observed mobilization activity among the *Saimiri, Cebus*, or *Sapajus* genera prompted us to construct a sequence alignment of all owl monkey lineage-specific Platy-1 elements (supplementary file 5 and fig. S1, Supplementary Material online).

**Fig. 5.**
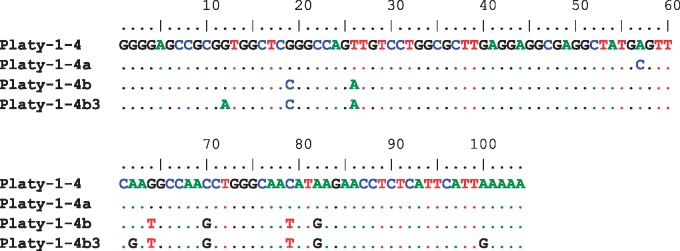
—Platy-1 consensus sequence alignment. The consensus sequences of Platy-1-4 and Platy-1-4a were aligned to the newly discovered 4b_*aotus* and 4b3_*aotus* subfamilies discovered via an alignment of all of the full-length Platy-1 elements ascertained from the owl monkey genome. Dots represent a shared nucleotide while diagnostic substitutions are shown as the corrected base.

After comparing owl monkey-specific loci to the Platy-1-4 consensus sequence reported in Konkel et al. (2016), at least two distinct diagnostic mutations occurred since *Aotus* diverged from the other genera. Among the loci present in the owl monkey genome, there were multiple shared diagnostic mutations at positions 19 (G to C transversion), 26 (T to A transversion), 64 (G to T transversion), 70 (C to G transversion), 79 (C to T transition), and 82 (A to G transition). This newly discovered subfamily was named Platy-1-4b_*aotus* (*n* = 58) and is aligned in (fig. 5). The nomenclature convention is as follows: this subfamily appears to have derived from Platy-1-4 but had different diagnostic substitutions than Platy-1-4a and was discovered in owl monkey.

Another diagnostic change was identified among *Aotus-*specific loci, some of which were fixed present while others were polymorphic. While sharing all diagnostic mutations that compose Platy-1-4b_*aotus*, there were three additional diagnostic mutations at positions 12 (G to A transition), 62 (A to G transition), and 100 (A to G transition). This new subfamily was termed Platy-1-4b3_*aotus* (*n* = 10). This follows standard nomenclature for naming repeats, as this subfamily has all the mutations of Platy-1-4b_*aotus* with 3 additional mutations (Batzer et al. 1996; fig. 5). Platy-1 mobilization in the *Aotus* lineage is consistent with the stealth model of SINE amplification dynamics (Han et al. 2005b) in which a few very old elements remain dormant for millions of years before slowly emerging with active daughter elements.

In the owl, squirrel, and capuchin monkey genomes analyzed in this study, no Platy-1 subfamilies younger than Platy-1-1 to Platy-1-6 were identified in the initial RepeatMasker analysis. This is in contrast to the marmoset genome where 62 subfamilies were discovered and all are present in [calJac3] (Konkel et al. 2016). As a part of that initial marmoset study, a subset of Platy-1 elements representing the majority (50 of 62) of subfamilies were analyzed by PCR to assess their distribution among NWM species. A graphic illustration of those results is shown in: (supplementary file 5 and fig. S2*a*, Supplementary Material online). Subfamilies shared among all NWM on this graph belonged to the oldest subfamilies (Platy-1-1 through Platy-1-3) and one insertion specific to all Cebidae belonged to subfamily Platy-1-4a. These data are in agreement with the RepeatMasker analysis performed in this study that identified the subfamily range between 4 and 4a as the source of lineage-specific elements reported here.

### Divergence of Platy-1 Subfamilies in NWM

**Fig. 6.**
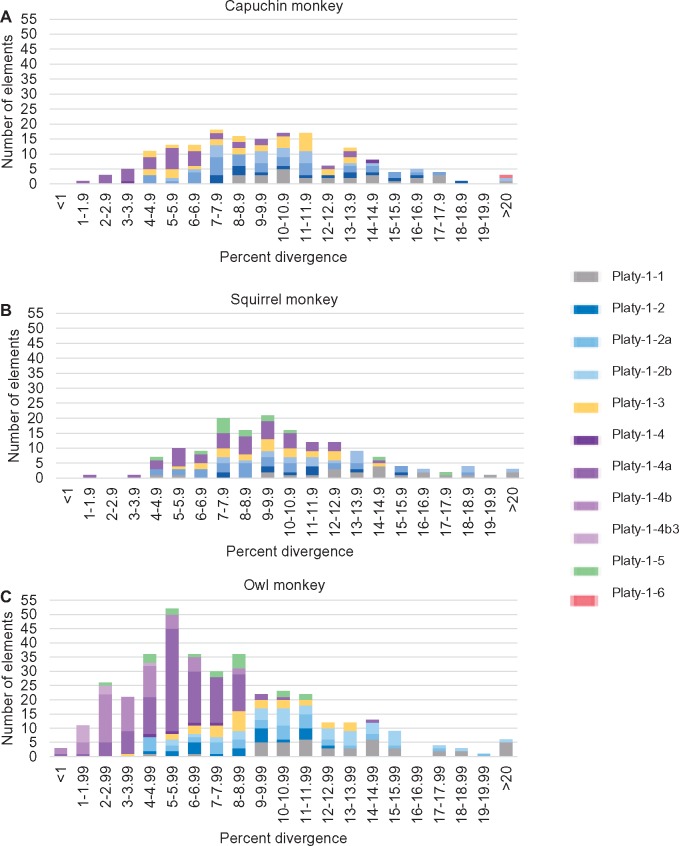
—Platy-1 nucleotide divergence rates. Percent divergence from the subfamily consensus sequence of full-length Platy-1 elements. (*A*) Capuchin monkey Platy-1 elements. (*B*) Squirrel monkey Platy-1 elements. (*C*) Owl monkey Platy-1 elements. The percent divergence was recorded from the RepeatMasker output.

Among the Platy-1 subfamilies there is a wide range of nucleotide divergence values from the respective subfamily consensus sequences as identified by RepeatMasker (fig. 6). A higher percent divergence from the respective subfamily consensus sequence is considered generally indicative of the age of the insertion event, as older elements have more time to accumulate random mutations. Mobile elements, on average, accumulate mutations at a neutral rate. As a consequence, the divergence can be used as an estimate of the age of the insertion. Figure 6 illustrates that the vast majority of the full-length Platy-1 elements identified in this study are relatively old. This finding is consistent with the PCR results from the squirrel and capuchin monkey lineage-specific DNA panels in which all the lineage-specific insertions had reached fixation throughout the genus. The average age corresponding with the percent divergence of the predicted lineage-specific Platy-1 insertions reported for the capuchin monkey is 12.4 Ma with a range from 4.8 to 22 Ma, while the average age of the lineage-specific Platy-1 insertions reported for squirrel monkey is 13.2 Ma with a range from 3.2 to 27.7 Ma. Older Platy-1 subfamilies correspond with higher percent divergence and therefore higher average age (e.g., capuchin monkey and squirrel monkey Platy-1-1: 21.8 and 23.4 Ma, respectively) (supplementary file 4, Supplementary Material online). This finding is in sharp contrast to the marmoset genome in which nearly 10% (224/2,268) of the Platy-1 elements reported were nearly identical to their respective consensus sequences and almost 25% had a percent divergence of 1.5% or less (Konkel et al. 2016). However, the nucleotide divergences calculated for the older Platy-1 subfamilies discovered in the common marmoset genome are in agreement with the divergence estimates gleaned from the data in this study (supplementary file 5 and fig. S2*b*, Supplementary Material online).

The lower nucleotide divergence values of the Platy-1 insertions found in the owl monkey genome (fig. 6*C*) were consistent with more recent insertions and are in agreement with the polymorphic loci found via PCR. The average age of the predicted lineage-specific owl monkey Platy-1 insertions calculated from the percent divergence of the insertion sequence to its respective consensus sequence is 8.5 Ma, with a range from 0 to 25.4 Ma (supplementary file 4, Supplementary Material online).

It is of note that these age estimates are based on a retrotransposable element that is only ∼100 bp in length and therefore could represent a fairly broad range. Even a one nucleotide change is equivalent to 1% divergence or ∼1.66 Myr. However, these results are intended to emphasize that the NWM genomes studied here contain primarily older Platy-1 elements, as compared with the marmoset genome in which the relative divergence values and age estimates illustrate that the marmoset genome contains large quantities of younger elements.

### Shared Platy-1 Elements

Over half (127/230; 55%) of the shared elements identified using a mugsy alignment (see Materials and Methods) were found within all four NWM genomes analyzed (marmoset, owl monkey, squirrel monkey, and capuchin monkey). The actual number is likely higher than this data set reflects due to lack of homology across multiple genome assemblies. These data are in agreement with the low number of lineage-specific insertions found in the NWM genomes analyzed in this study.

Five of the loci that were predicted to be lineage-specific in the squirrel monkey and capuchin monkey genomes were experimentally determined by PCR to be shared between the aforementioned genomes. These five loci were fixed present in all individuals representing the *Saimiri*, *Cebus*, and *Sapajus* genera (supplementary files 1 and 3, Supplementary Material online). These data are consistent with the close established relationship between *Saimiri*, *Cebus*, and *Sapajus*. In addition, these shared elements all belonged to the 4a Platy-1 subfamily, indicating that the age of these elements could reflect the evolutionary divergence time of *Saimiri*, *Cebus*, and *Sapajus* from other Cebids.

## Discussion

This study expanded upon the research reported by Konkel et al. (2016) by not only recovering Platy-1 insertions unique to other NWM genomes but also analyzing the amplification dynamics of these insertions. It is striking to note that there are a considerably lower number of Platy-1 repeats in owl, capuchin, and squirrel monkeys compared with the expansion and proliferation of Platy-1 insertions seen in the marmoset genome (Konkel et al. 2016). However, when comparing the three NWM genomes included in this study, there is a larger number of total, full-length, and linage-specific insertions found in the owl monkey genome than in the capuchin and squirrel monkey genomes (table 2). Platy-1 mobilization in owl monkeys appears to have been relatively quiescent for millions of years, dating back to the 4a subfamily, and only recently resumed with modest retrotransposition activity leading to the origin of two new *Aotus* lineage-specific subfamilies. By contrast, Platy-1 retrotransposition in capuchin and squirrel monkeys remains quiescent. One possible explanation is polymorphic loci were subject to lineage sorting during speciation, potentially eliminating source drivers for Platy-1 mobilization. This explanation is consistent with the lower overall numbers of Platy-1 elements in the capuchin and squirrel monkey genomes and higher overall percent divergences of the elements from their consensus sequences. In addition, all of the lineage-specific loci ascertained from the capuchin and squirrel monkey genomes were determined to be fixed present. These data indicate negligible recent Platy-1 mobilization in these lineages. This slow propagation is likely not due to a lack of available enzymatic machinery as it has been shown that L1, the element that provides the necessary enzymes for TPRT, has recently amplified in *Saimiri* among other NWM species (Feng et al. 1996; Boissinot et al. 2004).

These findings suggest that the extensive proliferation of Platy-1 elements in the common marmoset is the exception, rather than the norm in NWM genomes. Such disparities could be the result of differing effective population sizes after speciation, opposing environmental pressures, or genomic environment of the Platy-1 insertions in the different genera. There are also biological differences that might play a role. For example, marmosets have a unique aspect to their reproduction in that they mostly produce twins. The twinning of marmoset leads to genetic chimeras. This inherent genetic diversity in addition to the rapid reproduction of marmosets may have led to an environment favorable to retrotransposable element propagation. (Consortium 2014; Harris et al. 2014). The peak rate of Platy-1 propagation reportedly occurred with the rise of the marmoset ancestor (Konkel et al. 2016) when several Platy-1 subfamilies were active in parallel. In contrast, early Platy-1 evolution likely started with a low number of source elements resulting in very slow mobilization as illustrated by the NWM lineages analyzed in this study.

The polymorphic loci identified in this study that delineate between red- and gray-necked owl monkeys may be particularly useful for medical studies in which species identification is important. Owl monkeys have long been used as an animal model for malaria, with *Aotus lemurinus griseimembra* as the primary species used that is susceptible to the parasite responsible for causing this particular human malady (Herrera et al. 2002; Moreno-Pérez et al. 2017). While other owl monkey species have varying degrees of malaria susceptibility, the results with *A. l. griseimembra* have been particularly reproducible. Another owl monkey species, *A. vociferans*, is also susceptible to infection, but not as widely used as *A. l. griseimembra.* It is of note that both of these species are considered gray-necked (Herrera et al. 2002). Having reproducible genetic markers to distinguish between gray- and red-necked owl monkeys may be of value for biomedical studies. Although only a few polymorphic loci were identified in the owl monkey genome, with a small fraction of those showing a clear separation between gray- and red-necked owl monkeys, these markers provide a quick, simple, and unambiguous identification that is not currently available for this organism.

Platy-1 insertions observed in this study were often flanked by an *Alu* on either the 5′ or 3′ end of the Platy-1 element, and on rare occasions both sides. As reported by Konkel et al. (2016), Platy-1 elements with intact TSDs also possess endonuclease cleavage sites, indicating that Platy-1 elements have the same sequence and/or insertional preference as *Alu* elements.

Although Platy-1 elements may occasionally highjack *Alu* movement, Platy-1 elements are present in substantially lower overall numbers than *Alu* insertions in NWM. For example, a recent study of the *Saimiri* genome determined that there were 739,636 full-length and 43,201 lineage-specific *Alu* elements (Baker et al. 2017), a much higher number than the full-length and lineage-specific Platy-1 elements analyzed in this study. In addition, the majority of the Platy-1 elements characterized in this study were shared among all four of the NWM genomes studied. This indicates that there are simply not enough phylogenetically informative Platy-1 elements to be able to resolve NWM relationships. *Alu* elements may provide the key to elucidating NWM relationships as they have previously resolved difficult primate connections (Ray et al. 2005; McLain et al. 2012, 2013; Meyer et al. 2012; Walker et al. 2017; Jordan et al. 2018). A whole genome comparison of *Alu* insertion polymorphisms among the four NWM genomes described here may prove useful for elucidating some of the NWM relationships.

It is important to note that the repeats identified in this study were ascertained from the reference genome for all of the species studied. In addition, loci that were analyzed via PCR needed to be in conserved regions of all four genomes for confirmation of lineage-specificity. Sufficient time may have also passed that some of the insertions belonging to older subfamilies have experienced sufficient decay and were not recognized by RepeatMasker in the initial genome screening. It is therefore possible that the number of lineage-specific repeats and overall Platy-1 content in the NWM genomes analyzed is somewhat higher than reported. Undoubtedly, all three genomes have a sharply lower number of Platy-1 elements compared with marmoset.

## Supplementary Material

Supplementary data are available at *Genome Biology and Evolution* online.

## Supplementary Material

Supplementary Data
