# Supplementary material for: Amplification Dynamics of Platy-1 Retrotransposons in the Cebidae Platyrrhine Lineage
Source: Genome Biol Evol. 2019 Mar 19;11(4):1105–16. doi: 10.1093/gbe/evz062 (PMC6464705; doi:10.1093/gbe/evz062)
Supplement: Supplementary Data [file evz062_supp.zip › Supplementary File 5.docx]

10 20 30 40 50 60

....|....|....|....|....|....|....|....|....|....|....|....|

**Platy-1-1_NW_012163313.1:49684** **---------AGGCGC-AGTGGCTCAGGC-----CT--ATTGTCTTG----GCACGT----**

**Platy-1-1_NW_012163535.1:17840** **------GGG..CT..-.A..........-----.A--G.....C.A----.TG.A.----**

**Platy-1-1_NW_012162423.1:29766** **-------GG..C.A.-G.........A.-----.A--C.....C..----....----AT**

**Platy-1-1_NW_012163535.1:32779** **------GGG...T..-CA..........-----..--GCA...C..----.AG.A.----**

**Platy-1-1_NW_012163536.1:46396** **-------GG..C.AT-G......A....-----..--G.....C..----.AG.------**

**Platy-1-1_NW_012169090.1:29360** **------GGG..C...-............-----.A--G.....C..----.TG.C.----**

**Platy-1-1_NW_012170313.1:34330** **------AGG..CT.T-G..A........-----.A--G.....C..----....----AT**

**Platy-1-1_NW_012170424.1:19233** **-----AGGG..C.AT-G...........-----..--G.....C..----..G.A.----**

**Platy-1-1_NW_012171090.1:58916** **------GGG..CT..-.....T.T....-----..--G.....C..----....----AT**

**Platy-1-1_NW_012171312.1:20970** **-------GG..C..T-G.........TT-----..--G.....C..----.AG.----AT**

**Platy-1-1_NW_012171312.1:22155** **------GGG....A.-G...........-----..--G.....C..----.TGAAG----**

**Platy-1-1_NW_012172090.1:24950** **-------GG..C.AA-.......G....-----..--G.....C..----..G.----AT**

**Platy-1-1_NW_012173534.1:35510** **---CTAGGG..C.A.-G...........-----.A--G.....C..----.TG.A.----**

**Platy-1-1_NW_012175091.1:28244** **------GGG..C.T.-............-----.C--T..C..C..----..G...----**

**Platy-1-1_NW_012176869.1:33712** **-------GG..CT..-G...........-----..--G.....C..----.TG.A.----**

**Platy-1-1_NW_012177091.1:48611** **-------GG..CT..-.....G......-----.A--G.......A----....A.----**

**Platy-1-1_NW_012177646.1:34834** **--------G..C.A.-............-----.A--G.....C.T----.TG.A.----**

**Platy-1-1_NW_012177979.1:10635** **------GGG.TC..A-.......G....-----.A--......C..----..G.A.----**

**Platy-1-1_NW_012178646.1:26805** **----GAGGG..CT.T-G...........-----.A--G.....C..----.TG.A.----**

**Platy-1-1_NW_012183958.1:14675** **------GGGT.TG..-.A..........-----.C--G..A..C..----..G.----AT**

**Platy-1-1_NW_012184014.1:30269** **----TTGGG..C..T-G...........-----.G--G.....C..----..G.A.----**

**Platy-1-1_NW_012184214.1:36295** **-------AG.CC.A.-G...........-----..--G.....CA.----....----AT**

**Platy-1-1_NW_012184536.1:15341** **---------..CT.T-G........A..-----.A--G.....C..----.T..----AT**

**Platy-1-1_NW_012184613.1:35624** **-----------TT..-.......T....-----.A--G.....C..----.TG.A.----**

**Platy-1-1_NW_012184646.1:76762** **------GAG..CT..-G...........-----.C--......C..----.TGT----AT**

**Platy-1-1_NW_012184647.1:17260** **------GGG..A...-..........AT-----..--G.....C..----.TG.A.----**

**Platy-1-1_NW_012184746.1:45925** **-------GG..CTTT-GC...G......-----..--GC....CG.----.TGT----AT**

**Platy-1-1_NW_012184979.1:12918** **-------GG..CT..-G...........-----..--G.....C..----....----AT**

**Platy-1-1_NW_012185580.1:14037** **------GTG..C.A.-C...........-----..--G.....C..----....----AT**

**Platy-1-1_NW_012185646.1:80324** **------GGG..C.TT-G...........-----.A--G.....C..----..TT..----**

**Platy-1-1_NW_012186090.1:22025** **---------....AT-GC..A....CA.-----..--......C..----......C--T**

**Platy-1-1_NW_012186313.1:66308** **---------....AT-G........AT.-----.C--T.....C..----....A.----**

**Platy-1-1_NW_012187646.1:68445** **----CTGGG...TA.-G...........-----..--G.G...C..----.A.AAC----**

**Platy-1-1_NW_012187757.1:36641** **-------GG.....T-G...........-----..--..G..TC..----.TG.A.----**

**Platy-1-1_NW_012187946.1:15918** **------GGG..T...-...A.......T-----..--G.....C..----..C.A.----**

**Platy-1-1_NW_012188868.1:11813** **------GGG..CT..-G...........-----..--G.....C..-----...----AT**

**Platy-1-1_NW_012189090.1:4979-** **-------GGG.CT..-............------A--G.....C..----..G.A.----**

**Platy-1-1_NW_012189423.1:15745** **-----GGGG...T.G-.A..........-----.A--G........----.TG.A.----**

**Platy-1-1_NW_012189423.1:49188** **----GGGGG...T.T-G...........-----..--G.....C..----.AG.A.----**

**Platy-1-1_NW_012189424.1:43236** **----AAGGG..CA.T-G...........-----.A--G.....C..----..T.A.----**

**Platy-1-1_NW_012190534.1:27184** **---------..CT..-G...........-----.A--......C..----..G.----AT**

**Platy-1-1_NW_012190979.1:22614** **-------GG....TT-G...........-----.A--G.....C..----...T..----**

**Platy-1-2_NW_012164091.1:26806** **-------GG..C.A.-GA..........-----.A--G.....C..----....----TT**

**Platy-1-2_NW_012164091.1:26906** **------GGG..C.A.-GA..........-----.A--G.....C..----....----TT**

**Platy-1-2_NW_012165424.1:13538** **----------.C.A.-CA..........-----.A--G.....C..----..G.----TT**

**Platy-1-2_NW_012169090.1:16710** **-------GG..C.AA-G...........-----.A--G.....C..----..C.----TT**

**Platy-1-2_NW_012169535.1:14903** **--------G..C.AA-G...........-----.A--G.....C..----.TG.----TT**

**Platy-1-2_NW_012172423.1:21747** **-------GG..C.AT-G...........ACTGA..--G.....C..----.TG.----TT**

**Platy-1-2_NW_012172535.1:90777** **---------..C.A.-............-----.A--G.....A..----..G.----TG**

**Platy-1-2_NW_012173534.1:79384** **----------.C.C.-CA......C...-----.A--G.....C..----..G.----TT**

**Platy-1-2_NW_012176424.1:26496** **-------GGG.C.A.-G..A........-----.A--G..C..C..----.AG.----TT**

**Platy-1-2_NW_012184158.1:19413** **---CAAAGG..C.A.-...A........-----.A--G.....C..----....----TT**

**Platy-1-2_NW_012184202.1:16754** **-----AGGG..C.A.-G...........-----.A--GC....CA.----..G.----TT**

**Platy-1-2_NW_012184980.1:57576** **-----AGGG..C.A.-G...........-----.A--G.....C..----.TG.----TT**

**Platy-1-2_NW_012185302.1:16119** **------GGGT.C.A.-G..T........-----.A--G.....C..----..T.----TT**

**Platy-1-2_NW_012187201.1:60133** **-------GG..C.A.-............-----.A--C.....C..----....----AT**

**Platy-1-2_NW_012187202.1:21081** **------GGT..C.A.-............-----.A--G.....C..----....----AT**

**Platy-1-2_NW_012187325.1:43653** **---------..C.A.-............-----.A--G.....C..----....----TT**

**Platy-1-2_NW_012188646.1:21539** **--------A..C.A.-C...T.......-----.A--G.....C..----.TG.----CT**

**Platy-1-2_NW_012189423.1:10665** **----ATGGG..C.A.-.......-....-----..--G.....C..----..G.----TT**

**Platy-1-2_NW_012189423.1:12584** **------GGG..C.A.-G...........-----.A--G.....C..----.TG.----TT**

**Platy-1-2_NW_012189424.1:90770** **---TATGGG..C.A.-.T..........-----.A--G.CA.....----..C.----TT**

**Platy-1-2_NW_012189757.1:60581** **-------GA..C.A.-...A........-----.A--G.....CA.----....----TT**

**Platy-1-2_NW_012190868.1:93211** **-------GG..C.A.-G...........-----.A--G.....C..----..G.----TT**

**Platy-1-2a_NW_012162647.1:5295** **-------GA..C..T-G...........-----.A--G.....C..----.TG.----TT**

**Platy-1-2a_NW_012163869.1:6833** **-------GG..C.A.-............-----.A--G.....C..----....----TT**

**Platy-1-2a_NW_012164202.1:3713** **-GATGCGGG..C...-G........CT.-----GA--G.....C..----..G.----TT**

**Platy-1-2a_NW_012164424.1:6158** **---------.ACT..-............-----.A--......C..----....----TT**

**Platy-1-2a_NW_012165424.1:1861** **-------GG..CT..-C...........-----TA--G...-----------------TT**

**Platy-1-2a_NW_012167424.1:7138** **------GGG..C.A.-............-----.A--G.....C..----.T..----TT**

**Platy-1-2a_NW_012167646.1:1017** **---TCTGGG..CA..-G......TG...-----.A--G.....C..----....----TT**

**Platy-1-2a_NW_012167868.1:8952** **----CGGGG..CT..-............-----.A--G.....C..----.TG.----TT**

**Platy-1-2a_NW_012170868.1:3906** **-----------C..A-G...........-----.A--G.....C..----.GG.----TT**

**Platy-1-2a_NW_012171312.1:1107** **-------GG..C.A.-G...........-----.A--G...----------TG.----TT**

**Platy-1-2a_NW_012172535.1:1491** **----------.C.A.-T...........-----.A--G.....C..----....----TG**

**Platy-1-2a_NW_012173534.1:4251** **-------GG..C.A.-...A........-----.A--G.....C..----.TG.----TT**

**Platy-1-2a_NW_012173535.1:9675** **-----TGGG..CT..-............-----.A--GG.......----....----TT**

**Platy-1-2a_NW_012176424.1:1014** **------GGG..C...-............-----.A--G.....C..----....----TT**

**Platy-1-2a_NW_012176868.1:1095** **------GGA..C.A.-G...........------A--G.....C..----.TGT----GT**

**Platy-1-2a_NW_012177979.1:9460** **---------..CT..-............-----.A--G.A...C..----....----TT**

**Platy-1-2a_NW_012178424.1:8756** **------GGG..C.A.-.A..........-----.A--G..T..C-A----....----TT**

**Platy-1-2a_NW_012179535.1:1101** **-------GG..CT..-CA..........-----.A--GC....C..----..G.----TT**

**Platy-1-2a_NW_012183869.1:1597** **-----GGGG..C.A.-............-----.A--G.....A..----....----TT**

**Platy-1-2a_NW_012184025.1:1425** **------GGG..C...-T...........-----.A--G....TC..----.TG.----TT**

**Platy-1-2a_NW_012184091.1:5783** **-------GG..CA..-CA..........-----.A--G.....C..----.TG.----TT**

**Platy-1-2a_NW_012184202.1:1281** **-----GAAG..C...-G...........-----TG--..A...C..----.TG.----TT**

**Platy-1-2a_NW_012184358.1:8743** **-------GG..CT..-G..........T-----.A--G.A.AACC.----.TG.----TT**

**Platy-1-2a_NW_012184757.1:4571** **---------..CT.T-G.......C...-----.A--G.....C..----..G.----TT**

**Platy-1-2a_NW_012184802.1:5276** **-------GGG.C...-..........A.-----.A--G.....C..----.TG.----TT**

**Platy-1-2a_NW_012184857.1:1202** **------GGG..CT.A-G......T..A.-----.A--GC....C..----.AG.----TT**

**Platy-1-2a_NW_012184868.1:5356** **---------..CT..-............-----.A--G.....C..----..G.----TT**

**Platy-1-2a_NW_012185025.1:8158** **-------GG..CT..-T...........-----.A--G.....C..----.TG.----TT**

**Platy-1-2a_NW_012185313.1:4572** **-------GG..C.A.-G...........-----.A--G.....C..----.TG.----TT**

**Platy-1-2a_NW_012186868.1:3979** **--------G..C.T.-C....T......-----.A--G.....C..----.AGA----TT**

**Platy-1-2a_NW_012187269.1:8608** **----CTGGG..C...-G...........-----.A--G.....C..----..G.----TT**

**Platy-1-2a_NW_012187424.1:6175** **-----------C..T-.A..........-----.A--......C..----....----TT**

**Platy-1-2a_NW_012188312.1:2203** **--------G..C...-C......--------------------C..----....----AT**

**Platy-1-2a_NW_012188646.1:2664** **-------GG.CCT.T-G....T......-----.A--......C..----.TG.----TT**

**Platy-1-2a_NW_012189312.1:9461** **--------G.ACT..-............-----.A--G...C.C..----....----TT**

**Platy-1-2a_NW_012189423.1:1252** **------GGG..CT..-G...........-----.A--G.....C..----.TGGTG-GTT**

**Platy-1-2a_NW_012191312.1:2658** **------GGG..C.T.-G...........-----.A--G.....C.A----....----TT**

**Platy-1-2b_NW_012162425.1:6193** **-------GG....AT-G...C.C.....-----..--G.G...C.A----....C.----**

**Platy-1-2b_NW_012165757.1:1879** **------GGG..C.T.-G...T.......-----..--G.....C..----...T----AT**

**Platy-1-2b_NW_012165757.1:2715** **---------..CG..-G....T.....T-----.A--G.....C..----.TG.----TT**

**Platy-1-2b_NW_012165980.1:1672** **--------A..CT..-............-----.A--G.....C..----....----AT**

**Platy-1-2b_NW_012166091.1:5131** **-------GG..TT..-............-----.A--G.....C..----....A.----**

**Platy-1-2b_NW_012166313.1:3251** **--------G..CT.T-G..A........-----..--G.....C.A----.TG.A.----**

**Platy-1-2b_NW_012166535.1:5728** **--------G..CT.A-G...T.......-----.A--G.....C.A----C...----AT**

**Platy-1-2b_NW_012166868.1:4843** **-------GG..AA..-.A.........T-----.A--...C..C..----....----TT**

**Platy-1-2b_NW_012167202.1:5072** **-------GA..C...-T...........-----.A--G.....C..----..G.----TT**

**Platy-1-2b_NW_012167313.1:2947** **-------GG..T.AT-G...........-----TA--G.....C..----....----AT**

**Platy-1-2b_NW_012169313.1:3109** **-------GG..CTAT-G.........C.-----..--G.....C..----.TG.----AT**

**Platy-1-2b_NW_012172201.1:9584** **----TAGGG..C.AT-T..........T-----.A--G.GT..C..----....----TT**

**Platy-1-2b_NW_012173534.1:2304** **-------GG..C.AT-G...A..T....-----.A--G.....C..----..G.----AT**

**Platy-1-2b_NW_012173534.1:2643** **------GGGTA..AT-G.........T.-----T.-----...C..----.TG.A.----**

**Platy-1-2b_NW_012173535.1:2973** **------GGG..CT.T-G.......C...-----..--G.....C..----....----AC**

**Platy-1-2b_NW_012174646.1:7972** **--------G..C.A.-...C........-----..--.C....C..----.TG.----TT**

**Platy-1-2b_NW_012175796.1:638-** **-------GGTA..AT-G.........T.-----T.-----...C..----.TG.A.----**

**Platy-1-2b_NW_012180543.1:1009** **------GGG..CT..-G..........T-----..--G..TC.C..----.TG.----TT**

**Platy-1-2b_NW_012180868.1:6439** **---TGGGGG..T.A.-......C-----------A--G.....C..----....----TT**

**Platy-1-2b_NW_012181646.1:7893** **-----TGGG..C.A.-...A..C.C...-----.A--C.....C..----.TG.----GT**

**Platy-1-2b_NW_012182423.1:7347** **--TCCTGGG..C..T-G...........-----.A--G.....C..----.TG.----TT**

**Platy-1-2b_NW_012183534.1:1116** **CATTACAGG.ACT.T-G...........-----..--G...C.C..----....----AT**

**Platy-1-2b_NW_012183534.1:1895** **---------.ACT..-..........C.-----.A--G........----A...----TT**

**Platy-1-2b_NW_012183868.1:7418** **-------GG..C.A.-............-----.A--G.....C..----.TG.T.----**

**Platy-1-2b_NW_012183869.1:1188** **----TAGGG..C...-T...........-----.A--G.-...C..----..G.----AT**

**Platy-1-2b_NW_012184424.1:3175** **----------.T.A.-G.......C...-----..---.....C..----.AG.----TT**

**Platy-1-2b_NW_012185114.1:1027** **--------G..C.A.-G...........-----.A--G.....C-.----.TG.----TT**

**Platy-1-2b_NW_012186090.1:1197** **------GGG..CT..-..C.........-----.A--GC....C..----..G.----TT**

**Platy-1-2b_NW_012186202.1:4485** **-------GG..CT.T-G...........-----.A--G.....CA.----.TG.----TT**

**Platy-1-2b_NW_012186313.1:7737** **-------GGG.C.A.-.........AT.-----..--G....TC..----....----TT**

**Platy-1-2b_NW_012186424.1:3482** **-------GG.AC.C.-............-----.A--G.....C..----..G.----AT**

**Platy-1-2b_NW_012187424.1:5967** **------GGG..C.T.-T........C.T-----.A--G.....C..----....----TT**

**Platy-1-2b_NW_012188090.1:1455** **-------GG..CT.T-G..........T-----.A--G.....C..----A.G.----TT**

**Platy-1-2b_NW_012188090.1:1622** **--GATAGGG.AC..T-G...........-----..--G..T..CG.----.TG.C.----**

**Platy-1-2b_NW_012188646.1:1667** **----------.CT..-G........C..-----.A--G.....C..----....----TT**

**Platy-1-2b_NW_012188757.1:8687** **-----GGGGG.CT..-..........T.-----T.--G.....C..----..G.----TT**

**Platy-1-2b_NW_012189090.1:5283** **------GGG..C.A.-............-----.A--G.....C..----..G.----TT**

**Platy-1-2b_NW_012189423.1:1292** **-----TGAG..C...-T...........-----.A--G.....G..----A...----TT**

**Platy-1-2b_NW_012189757.1:6380** **---------..C...-G...........-----.A--G..AA.C..----.TG.----TT**

**Platy-1-2b_NW_012190534.1:2742** **-----GGGG..C...-G...........-----.A--G.....C..----..G.----AT**

**Platy-1-2b_NW_012190646.1:3121** **------GAG..C..T-G..........A-----.A--G.....CA.----..G.----TT**

**Platy-1-2b_NW_012191312.1:2401** **-----TGGG..CT..-G...........-----.A--G.....C..----....----TT**

**Platy-1-3_NW_012162423.1:14651** **--------G..C.AT-G...........-----.A--G.....C..----....----TT**

**Platy-1-3_NW_012163091.1:14454** **-----GGGG..CTA.-............-----.A--G.....C..----....----TT**

**Platy-1-3_NW_012163202.1:30198** **-------GG..CT..-...........A-----.A--G.....C..----..G.----TT**

**Platy-1-3_NW_012164869.1:60849** **-----CGGG..C.A.-GAG.........-----.A--G.....C..----....----TT**

**Platy-1-3_NW_012165198.1:5928-** **-----GGGG..C...-............-----.A--G.....C..----..G.----TT**

**Platy-1-3_NW_012165535.1:14696** **------GGG..C...-G...........-----.A--G.....C..----.TG.----TT**

**Platy-1-3_NW_012167535.1:56481** **-----GGGG..C...-............-----.A--G.....C..----.TG.----TT**

**Platy-1-3_NW_012170313.1:12544** **--------G..T.A.-............-----.A--G.....C..----.TG.----TT**

**Platy-1-3_NW_012170535.1:46745** **------GGG..C...-............-----.A--G.....C..----.TG.----TT**

**Platy-1-3_NW_012173534.1:14650** **-------GG..-.T.-......----------------.....C..----....----TT**

**Platy-1-3_NW_012173534.1:34177** **------GGG..CT..-G....A.A....-----.A--G.....C..----.TG.----TT**

**Platy-1-3_NW_012174202.1:58320** **----ATGGG..T...-............-----TA--G.....C..----.TC.----CT**

**Platy-1-3_NW_012174424.1:28994** **------GGG..C...-............-----.A--G.....C..----.TG.----TG**

**Platy-1-3_NW_012178646.1:42536** **---------..C.A.-............-----.A--G.....C..----....----TT**

**Platy-1-3_NW_012179646.1:13674** **---------..CT.T-G...........-----..--G.....C..----....----AT**

**Platy-1-3_NW_012179646.1:13765** **----------.CT.T-G...........-----..--G.....C..----....----AT**

**Platy-1-3_NW_012180757.1:22137** **---------..C.AT-G..........T-----.A--G.....C..----.AG.----AT**

**Platy-1-3_NW_012181868.1:10555** **-----TGGG..C...-............-----.A--G.....C..----....----TT**

**Platy-1-3_NW_012183892.1:21329** **--------G..C...-............-----.A--G.....C..----.TG.----TT**

**Platy-1-3_NW_012184003.1:21311** **------AGG..CT..-............-----.A--G..C..C..----...T----TT**

**Platy-1-3_NW_012184092.1:29931** **-------GG..CT..-C...........-----.A--G.....C..----A...----TT**

**Platy-1-3_NW_012184092.1:47861** **--------A..C...-.......A....-----.A--G.....C..----..G.----AT**

**Platy-1-3_NW_012185313.1:81785** **---ACAGGG..C.A.-.........T..-----.A--G.....C..----..G.----TT**

**Platy-1-3_NW_012185535.1:12734** **-------GG..CT..-T....T......-----.A--G.....C..----.AG.----TT**

**Platy-1-3_NW_012185757.1:66791** **------GGG..CT..-T...........-----.A--G.....C..----.TG.----TT**

**Platy-1-3_NW_012186720.1:3413-** **-------GG..C.A.-............-----.A--G.....C..----....----TT**

**Platy-1-3_NW_012186757.1:60120** **------GGG..C.T.-............-----..--......C..----....A.----**

**Platy-1-3_NW_012188090.1:38232** **--------G..C.A.-............-----.A--C........----...A----TT**

**Platy-1-3_NW_012188458.1:41468** **-------------.G-G.A.......-T-----GG--C.....C..----....----TT**

**Platy-1-3_NW_012191423.1:67979** **------GGG..CTT.-............-----.A--G.....C..----....----TT**

**Platy-1-4_NW_012173980.1:20137** **------------...-G...........-----.A--......C..----.TG.----TT**

**Platy-1-4_NW_012189423.1:11596** **-------GG..C...-G.......CA..-----.A--G.....C..----..G.----TT**

**Platy-1-4_NW_012189868.1:48523** **---CTAGGG..C...-G.......T...-----.A--G.....C..----..G.----TT**

**Platy-1-4_NW_012190201.1:56706** **-------GG..T.T.-............-----.A--G.....C..----..G.----TT**

**Platy-1-4a_NW_012162647.1:5343** **-----CGGG..C.A.-G.......CA..-----.A--G.....C..----..G.----TT**

**Platy-1-4a_NW_012162869.1:5364** **-------GG..C...-G.......C...-----.A--G.....C..----..G.----TT**

**Platy-1-4a_NW_012162869.1:5365** **-------GG..C...-G.......C...-----.A--G.....C..----.TG.----TT**

**Platy-1-4a_NW_012163158.1:1929** **------GGG..C..T-G.......CA..-----.A--G.....C..----..G.----TT**

**Platy-1-4a_NW_012163535.1:2382** **-----AGGG..C...-G.......C...-----.A--G.....C..----..G.----TT**

**Platy-1-4a_NW_012163980.1:1247** **--------G..C.A.-G.......C...-----.A--G.....C..----.TG.----TT**

**Platy-1-4a_NW_012164202.1:2548** **-------GG..C.A.-G.......G...-----.A--G.....C..----..G.----TT**

**Platy-1-4a_NW_012164424.1:2374** **-----GGGG..C...-G.......T...-----.A--G....TC..----.TG.----TT**

**Platy-1-4a_NW_012164646.1:1456** **----------.C...-C...........-----.A--G..A..C..----....----TT**

**Platy-1-4a_NW_012165091.1:1698** **------GGG..C..T-G.......G...-----.A--G.....C..----.TG.----TT**

**Platy-1-4a_NW_012165091.1:2771** **-----AGAGC.C..T-G.......T...-----.A--G.....C..----.TG.----TT**

**Platy-1-4a_NW_012165091.1:5564** **---TTTGGG..C...-G...........-----TA--G.....C.C----.TG.----TT**

**Platy-1-4a_NW_012165535.1:1152** **------GGG..CT.T-G.......C...-----.A--G.....C..----..G.----TT**

**Platy-1-4a_NW_012165757.1:4481** **------GGG..C..A-G.......G...-----.A--G.....C..----..G.----CT**

**Platy-1-4a_NW_012165757.1:4482** **------GGG..C..A-G.......G...-----.A--G.....C..----..G.----CT**

**Platy-1-4a_NW_012165757.1:8542** **---------T.C.T.-G.......CA..-----.A--G.....C..----.TG.----TT**

**Platy-1-4a_NW_012165980.1:3779** **-----GGGG..C...-G......AG...-----.A--G.....C..----..G.----TT**

**Platy-1-4a_NW_012166202.1:2027** **-------GG..C.A.-G.......G...-----.A--G.....C..----..G.----TT**

**Platy-1-4a_NW_012166646.1:3729** **------GGG..C.A.-G...........-----.A--G.....C..----..G.----TT**

**Platy-1-4a_NW_012167202.1:4991** **-------GG..C.T.-G.......C..A-----.G--G.....C..----..G.----TT**

**Platy-1-4a_NW_012167313.1:3173** **------GGG..CT..-G.......C...-----.A--G.....C..----..G.----TT**

**Platy-1-4a_NW_012167424.1:2226** **------GGG..C...-G.......C...-----.A--G.....C..----..G.----TT**

**Platy-1-4a_NW_012167868.1:6778** **-------GG..C..T-G.......C...-----.A--G.....C..----..G.----TT**

**Platy-1-4a_NW_012168313.1:1440** **--------G..C...-G.......C...-----.A--G.....C..----.TG.----TT**

**Platy-1-4a_NW_012168424.1:4326** **--------G..C.A.-G.......G...-----.A--G.....C..----..G.----TT**

**Platy-1-4a_NW_012168646.1:1504** **-------GG..C...-G.......CA..-----.A--G.....C..----..C.----TT**

**Platy-1-4a_NW_012168979.1:8062** **-------GG..C...-G.......G..T-----.A--G.....C..----..G.----TT**

**Platy-1-4a_NW_012169090.1:2106** **------GGG..C...-........G...-----.A--G.....C..----..G.----TT**

**Platy-1-4a_NW_012169090.1:4300** **-------GG..C...C..ACTT......-----.A--G.....C..----..G.----TT**

**Platy-1-4a_NW_012169091.1:2859** **------GGG..C...-.......TG...-----.A--G...A.C..----.TG.----TT**

**Platy-1-4a_NW_012169313.1:2654** **-------GG..C...-G.......G...-----.A--G.....C..----..G.----TT**

**Platy-1-4a_NW_012169535.1:7280** **---------..C...-G.......C...-----.A--G.....C..----....----TT**

**Platy-1-4a_NW_012169979.1:4186** **--ATGGAGG..C...-C...........-----TA--G.....C..----..G.----TT**

**Platy-1-4a_NW_012170201.1:1053** **------GGG..C.A.-............-----.A--G.....C..----..G.----TT**

**Platy-1-4a_NW_012171126.1:2521** **------GGG..CT..-........C...-----.A--G.....C..----..GT----TT**

**Platy-1-4a_NW_012171201.1:1320** **-----AGGA..C...-G.......G...-----.A--G.....C..----.TG.----TT**

**Platy-1-4a_NW_012171312.1:1877** **------GGG..C.AT-G......TG...-----.A--G.....C..----.TG.----TT**

**Platy-1-4a_NW_012171312.1:5539** **----TCGGG..C...-........C...-----.A--G.....C..----..G.----TT**

**Platy-1-4a_NW_012173201.1:4719** **-----TGGG..C...-G.......G...-----.A--G.....C..----..G.----TT**

**Platy-1-4a_NW_012173312.1:2571** **---CTTGGG..C...-G.......G...-----.A--G.....C..----.TG.----TT**

**Platy-1-4a_NW_012173758.1:3202** **-----TGGG..C..T-G.......C...-----.A--G.....C..----..G.----TT**

**Platy-1-4a_NW_012173758.1:3282** **---------..C...-G.......C...-----.A--G.....C..----..G.----TT**

**Platy-1-4a_NW_012174424.1:7477** **---------..CT..-G.......C...-----.A--G.....C..----..G.----TT**

**Platy-1-4a_NW_012174646.1:1101** **--------G..CT..-G.......C...-----.A--G.....C..----..G.----TT**

**Platy-1-4a_NW_012175646.1:4412** **------GGG..C...-G.......C...-----.A--G.....C..----..G.----TT**

**Platy-1-4a_NW_012176202.1:1353** **-------GG..C...-........G...-----.A--G.....C..----....----TT**

**Platy-1-4a_NW_012176313.1:3041** **-------GG..C...-G.......G...-----.A--G.....C..----....----TT**

**Platy-1-4a_NW_012176535.1:1424** **------GGG..C.A.-G.......G...-----.A--G.....C..----....----TT**

**Platy-1-4a_NW_012176646.1:1884** **----------A....-G.......G...-----.A--G.....C..----....----TT**

**Platy-1-4a_NW_012176757.1:2674** **-------GG..C.A.-G.......T..T-----.A--G.....C..----..G.----TT**

**Platy-1-4a_NW_012177979.1:2731** **--------G..C.AT-G.......G...-----.A--G.....C..----..G.----TT**

**Platy-1-4a_NW_012178025.1:1167** **-------GG..C...-G.......C...-----.A--G.....C..----.TG.----TT**

**Platy-1-4a_NW_012178091.1:1955** **--------G..C...-G.......C...-----.A--G.....C..----..G.----TT**

**Platy-1-4a_NW_012178535.1:1006** **-------GG..C...-........C...-----.A--G....TC..----.GG.----TT**

**Platy-1-4a_NW_012178757.1:4212** **-----TGGG..C...-G.......C...-----.A--G.....C..----.AG.----TT**

**Platy-1-4a_NW_012181312.1:9214** **----------.CG..-G.......C...-----.A--G.....C..----.TG.----TT**

**Platy-1-4a_NW_012182535.1:1154** **------GGG..C...-........C...-----.A--G.....C..----.AG.----TT**

**Platy-1-4a_NW_012183201.1:3224** **-------GG..C.T.-G.......C...-----.A--G.....C..----.GG.----TT**

**Platy-1-4a_NW_012183802.1:7811** **----GGGGG..C...-G.......C...-----.A--G.....C..----....----TT**

**Platy-1-4a_NW_012183813.1:5526** **------GGG..C...-G.......G...-----.A--G.....C..----..G.----TT**

**Platy-1-4a_NW_012183824.1:1101** **--------G..C..T-G.......C...-----.A--G.....C..----..G.----TT**

**Platy-1-4a_NW_012183869.1:1041** **------GGG..C...-........C...-----.A--G.....C..----..G.----TT**

**Platy-1-4a_NW_012183869.1:1360** **-------GG..C...-G.......C...-----.A--G.....C..----..G.----TT**

**Platy-1-4a_NW_012183869.1:5673** **-------GG..CT..-G.......T...-----.A--G.....C..----..G.----TT**

**Platy-1-4a_NW_012183881.1:1201** **-------GG..C...-.......TG.A.-----.A--G.....C..----..G.----TT**

**Platy-1-4a_NW_012183958.1:1156** **-------GG..CA..-G.......C...-----.A--G....TC..----....----TT**

**Platy-1-4a_NW_012183980.1:1098** **-----GGGGCAC.AT-.....T......-----.A--G.....C..----.TG.----TT**

**Platy-1-4a_NW_012184047.1:9856** **-----GAGG..C...-G...........-----.A--G.....C..----..G.----TT**

**Platy-1-4a_NW_012184147.1:3563** **-------GG..C...-........CAA.-----.A--G....TC..----..G.----TT**

**Platy-1-4a_NW_012184269.1:1219** **-----GGGG..C...-G.......C...-----.A--G.....C..----..G.----TT**

**Platy-1-4a_NW_012184480.1:8638** **------GGG..C...-G.......G...-----.A--G.....C..----.GGT----TT**

**Platy-1-4a_NW_012184535.1:3014** **--------G..CT..-G.......G...-----.A--G.....C..----..G.----TT**

**Platy-1-4a_NW_012184535.1:7443** **-------GG..C.A.-G...........-----.A--G..T..C..----..G.----TT**

**Platy-1-4a_NW_012184602.1:1032** **------GGG..C...-G.......T...-----.A--G.....C..----..G.----TT**

**Platy-1-4a_NW_012184702.1:2422** **-----GGGG..C...-G.......T...-----.A--G.....C..----.TG.----TT**

**Platy-1-4a_NW_012184735.1:1484** **-------GG..C...-............-----.A--G.....C..----.AG.----TT**

**Platy-1-4a_NW_012184902.1:5722** **---TACGGG..C...-G.....C.C...-----.A--G.....C..----..G.----TT**

**Platy-1-4a_NW_012184980.1:7322** **------AGG..C..T-G...........-----.A--G.....C..----..G.----TT**

**Platy-1-4a_NW_012185202.1:8626** **---------..C.A.-G......TG...-----.A--G.....C..----..G.----TT**

**Platy-1-4a_NW_012185247.1:6115** **--------G..C.T.-.......TG----------------..C..----....----TT**

**Platy-1-4a_NW_012185313.1:1077** **--------G..C..T-G.......C...-----.A--G.....C..----..G.----TT**

**Platy-1-4a_NW_012185313.1:9239** **--------G..C...-G.......C...-----.A--G.....C..----..G.----TT**

**Platy-1-4a_NW_012185424.1:2354** **---------..CT..-........G...-----.A--G.....C..----..G.----TT**

**Platy-1-4a_NW_012185425.1:7570** **-----------C.A.-........C...-----.A--G.....C..----.TG.----TT**

**Platy-1-4a_NW_012185458.1:2034** **-------GG..C..T-........G...-----.A--G.....C..----..G.----TT**

**Platy-1-4a_NW_012185757.1:1154** **----------.C...-G.......C...-----.A--G.....C..----..G.----TT**

**Platy-1-4a_NW_012185757.1:1242** **------GGG..C..T-G.......C...-----.A--G.....C..----.TG.----TT**

**Platy-1-4a_NW_012185913.1:1882** **-------GG..C...-G.......G...-----.A--G.....C..----..G.----TT**

**Platy-1-4a_NW_012185957.1:9479** **-----TGGG..C.A.-G.......G...-----.A--T.....C..----..G.----TT**

**Platy-1-4a_NW_012186091.1:6351** **-----GGGG..C..T-G.......C...-----.A--G.....C..----.TG.----TT**

**Platy-1-4a_NW_012186114.1:2462** **------GAG..C...-G.......G...-----.A--G.....C..----..G.----TT**

**Platy-1-4a_NW_012186313.1:4701** **-------GG..CT..-G.......T...-----.A--G.....C..----..GT----TT**

**Platy-1-4a_NW_012186535.1:5683** **----TGGGG..C...-G.......GA..-----.A--G.....C..----..T.----TT**

**Platy-1-4a_NW_012186646.1:6551** **------GGG..CT..-G.......G...-----.A--G.....C..----.TG.----TT**

**Platy-1-4a_NW_012186757.1:1166** **------GGG..T.A.-G.......C...-----.A--G.....C..----..G.----TT**

**Platy-1-4a_NW_012186757.1:1875** **---GGTGGG..C...-G.......G...-----.A--G.....C..----..G.----TT**

**Platy-1-4a_NW_012186979.1:9295** **-AAGCAGGG..C..T-GA......C...-----.A--G.....C..----..G.----TT**

**Platy-1-4a_NW_012187202.1:5070** **---------..C...-G.......G...-----.C--G.....C..----..GT----TT**

**Platy-1-4a_NW_012187757.1:6197** **------GGG..C.AT-G.......CA.A-----.A--G.....C..----.A..----TT**

**Platy-1-4a_NW_012188090.1:6532** **----------.C.AT-G.......C...-----.A--G.....C..----.TG.----TT**

**Platy-1-4a_NW_012188868.1:4269** **-------GG..C...-G.......C...-----.A--G.....C..----..G.----TT**

**Platy-1-4a_NW_012189201.1:1245** **--------G..C...-G.......CA..-----.A--G.....C..----....----TT**

**Platy-1-4a_NW_012189201.1:2745** **------GGG.AC...-G.......C...-----.A--G.....C..----..G.----TT**

**Platy-1-4a_NW_012189423.1:1301** **------GGG..C..T-G......GG...-----.A--G.....C..----.TG.----TT**

**Platy-1-4a_NW_012189423.1:2606** **------GGG.AC...-........C...-----.A--G.....C..----..G.----TT**

**Platy-1-4a_NW_012189424.1:1588** **------GGG..C...-........CA..-----.A--G.C...C..----..G.----TT**

**Platy-1-4a_NW_012189979.1:1974** **-------GG..C..T-G.......C...-----.A--G.....C..----.TG.----TT**

**Platy-1-4a_NW_012189979.1:3723** **--CTTGGGG..C...-G.......C...-----.A--G.....C..----..G.----TT**

**Platy-1-4a_NW_012190534.1:1765** **------GGG..C...-G..A....CA..-----.A--G.....C..----..G.----TT**

**Platy-1-4a_NW_012190757.1:6050** **-------GG..C...-G.......G...-----.A--G.....C..----.TG.----TT**

**Platy-1-4a_NW_012190868.1:3122** **--------G..C.A.-G.......C...-----.A--G.....C..----..G.----TT**

**Platy-1-4a_NW_012191090.1:1473** **--------G..C.A.-G.......CA..-----.A--G.....C..----..T.----TT**

**Platy-1-4a_NW_012191090.1:7220** **-----------C..T-G.......C...-----.A--G.....C..----..G.----TT**

**Platy-1-4a_NW_012191201.1:4096** **---------..CTT.-G.......C...-----.A--G.....C..----..G.----TT**

**Platy-1-4a_NW_012191423.1:5463** **-----TGGG..C.A.-G.......C...-----.A--G.....C..----..G.----TT**

**Platy-1-4b_NW_012162758.1:2572** **------GGG..C...-G.......C...-----.A--GA....C..----A.G.----TT**

**Platy-1-4b_NW_012162869.1:6381** **-------GG..C.A.-G.......CA..-----.A--GA....C..----..G.----TT**

**Platy-1-4b_NW_012163202.1:1994** **------GGG..T...-G..C....C...-----.A--GA....C..----..G.----TT**

**Platy-1-4b_NW_012163535.1:2530** **--------G..C...-G.......C...-----.A--G.....C..CCTC..G.----TT**

**Platy-1-4b_NW_012163535.1:2531** **----------.C...-G.......C...-----.A--G.....C..CCTC..G.----TT**

**Platy-1-4b_NW_012163535.1:8297** **------GGG..CT..-G...A...C...-----.A--GA....C..----..G.----TT**

**Platy-1-4b_NW_012163647.1:6366** **-------GG..T..T-G.......C...-----.A--G........----..G.----TT**

**Platy-1-4b_NW_012163926.1:5965** **-------GA..C...-G.......CA..-----.A--G.....C..----..G.----TT**

**Platy-1-4b_NW_012164646.1:9630** **-------GG..C...-G.....C.C...-----.A--GA....C..----.TG.----TT**

**Platy-1-4b_NW_012165757.1:5703** **--AACGAGG..C...-G..A....C...-----.A--GA....C..----..G.----TT**

**Platy-1-4b_NW_012166868.1:1567** **------GGG..C...-G.......C...-----.A--GA....C..----..G.----TT**

**Platy-1-4b_NW_012167979.1:1849** **------GGG..CT..-G.......C...-----.A--GA....C..----..G.----TT**

**Platy-1-4b_NW_012168979.1:4354** **-------GG..C..T-G.......C...-----.A--GA....C..----..GA----TT**

**Platy-1-4b_NW_012169202.1:2313** **------GGG..C...-G.......CA..-----.A--GA....C..----..G.----TT**

**Platy-1-4b_NW_012169202.1:2314** **------GGG..C..T-G.......C...-----.A--GA....C..----..G.----TT**

**Platy-1-4b_NW_012169313.1:3048** **------GGA..C...-G.......C...-----.A--GA....C..----..G.----TT**

**Platy-1-4b_NW_012172423.1:1435** **-------GG..C..T-G.......C...-----.A--G.....C.C----..G.----TT**

**Platy-1-4b_NW_012172846.1:88-1** **--------G..C.A.-G.......C...-----.A--GA....C..----..GT----TT**

**Platy-1-4b_NW_012173423.1:6003** **-------GG..C.A.-G.......C...-----.A--GA....C..----..G.----TT**

**Platy-1-4b_NW_012173534.1:2605** **-------GG..C...-........C...-----.A--GA....C..----.TG.----TT**

**Platy-1-4b_NW_012173536.1:2838** **------GGG..C...-G.......C...-----.A--GA.A..C..----....----TT**

**Platy-1-4b_NW_012174202.1:6446** **-------GG..CT..-G......TC...-----.A-------.C..----.TGG----TT**

**Platy-1-4b_NW_012175568.1:1042** **-------GG..C.A.-G.......C...-----.A--GA....C..----....----TT**

**Platy-1-4b_NW_012179090.1:4388** **-----TGGG..C...-G.......C.A.-----.A--GA....C..----....----TT**

**Platy-1-4b_NW_012179424.1:2256** **--------G..C...-........C...-----.A--GA....C..----.TG.----TT**

**Platy-1-4b_NW_012180424.1:1829** **----TGGGG..C.A.-G.......C...-----.A--GA....C..----..G.----TT**

**Platy-1-4b_NW_012181312.1:4593** **------GGG..C...-........C...-----.A--GA....C..----A.G.----TT**

**Platy-1-4b_NW_012181868.1:3187** **-------GG..C...-G.......C...-----.A--GA....C..----..G.----TT**

**Platy-1-4b_NW_012182423.1:1093** **--------G..C...-G..A....C...-----.A--GAC...C..----..G.----TT**

**Platy-1-4b_NW_012182979.1:1332** **-------GG..C...-G.......C...-----.A--GA....C..----..G.----TT**

**Platy-1-4b_NW_012184003.1:1762** **----TGAGG..C.A.-GA......T...-----.A--GA....C..----..G.----TT**

**Platy-1-4b_NW_012184313.1:9985** **-------GG..C...-T.......C...-----.A--GA....C..----..G.----TT**

**Platy-1-4b_NW_012184436.1:2031** **-------GG..C...-G.......CA..-----.A--GA....C..----..G.----TT**

**Platy-1-4b_NW_012184513.1:8005** **-------GG..C.A.-G.......C...-----.A--GA....C..----.TG.----TT**

**Platy-1-4b_NW_012184646.1:1081** **-----AGGG..C...-G.......C...-----.A--GA....C..----..G.----TT**

**Platy-1-4b_NW_012184868.1:5660** **------GGG..C...-G.......C...-----.A--GA....C..----....----TT**

**Platy-1-4b_NW_012185091.1:4580** **-------GG..C...-G.......C...-----.A--GA....C..----..G.----TT**

**Platy-1-4b_NW_012185202.1:3769** **------GGG..CT..-G.......C...-----.A--GA....C..----..G.----TT**

**Platy-1-4b_NW_012185424.1:3710** **----------.C.AT-G.......C...-----.A--GA....C..----..G.----TT**

**Platy-1-4b_NW_012185424.1:3916** **-------GG..C...-GA......C...-----.A--GA...TG..----....----TT**

**Platy-1-4b_NW_012185646.1:1492** **-----AGGG..C..T-G.......C...-----.A--GA....C..----..G.----TT**

**Platy-1-4b_NW_012185757.1:1106** **--GAGAGGG..C...-GA......C...-----.A--GA....C..----..G.----TT**

**Platy-1-4b_NW_012185868.1:3666** **------GGG..C.A.-G..A....CA..-----.A--GA....C..----..G.----TT**

**Platy-1-4b_NW_012186091.1:1130** **-------GG..C...-G.......C...-----.A--G.....C..----.TG.----TT**

**Platy-1-4b_NW_012186202.1:1115** **-------GG..C...-G.......C...-----.A--GA....C..----..G.----TT**

**Platy-1-4b_NW_012186535.1:9707** **--GGAGGGG..C...-G.......C...-----.A--G.....C..----....----TT**

**Platy-1-4b_NW_012186979.1:1006** **-------GG..C...-G.......C...-----.A--GA....C..----..G.----TT**

**Platy-1-4b_NW_012187313.1:1001** **------GGG..C...-G.......C...-----.A--GA....C..----.AG.----TT**

**Platy-1-4b_NW_012188312.1:6490** **------GGG..C...-TA......C...-----.A--GA....C..----..G.----TT**

**Platy-1-4b_NW_012188480.1:1196** **-------GG..CT..-........C...-----.A--GA....C..----..G.----TT**

**Platy-1-4b_NW_012189423.1:1089** **----ATGGG..C...-G.......C...-----.A--GA....C..----..G.----TT**

**Platy-1-4b_NW_012189423.1:1825** **-------GG..A...-G.......C...-----.A--GA....C..----..G.----TT**

**Platy-1-4b_NW_012189423.1:1887** **------GGG..C...-.A......C...-----.A--GA....C..----..G.----TT**

**Platy-1-4b_NW_012190080.1:706-** **-------GG.AC...-G...........-----.A--GA....C..----..G.----TT**

**Platy-1-4b_NW_012190534.1:9595** **----TTGGG..C.A.-G.......C...-----.A--GA....C..----..G.----TT**

**Platy-1-4b_NW_012190868.1:1499** **-------GG..C..T-GA......T...-----.A--GA....C..----..G.----TT**

**Platy-1-4b_NW_012191201.1:6367** **------GGG..CTA.-G.......C...-----.A--GA....C..----.TG.----TT**

**Platy-1-4b_NW_012191312.1:2418** **----------.C...-G.......C...-----.A--GA....C..----..G.----TT**

**Platy-1-4b3_NW_012172535.1:174** **------GGG..C...-GA......C...-----.A--GA....C..----.TG.----TT**

**Platy-1-4b3_NW_012180979.1:316** **--------G..C...-GA......C...-----.A--GA....C..----..G.----TT**

**Platy-1-4b3_NW_012181312.1:304** **-------GG..CT..-GA......T...-----.A--GA....C..----..G.----TT**

**Platy-1-4b3_NW_012184424.1:305** **------GGG..C...-GA......C...-----.A--GA....C..----..G.----TT**

**Platy-1-4b3_NW_012184591.1:794** **--------G..C...-GA......C...-----.A--GA....C..----..G.----TT**

**Platy-1-4b3_NW_012184868.1:557** **------GGG..C...-GA......T...-----.A--GA....C..----..G.----TT**

**Platy-1-4b3_NW_012185158.1:100** **------GGG..C...-GA......C...-----.A--GA....C.A----.TG.----TT**

**Platy-1-4b3_NW_012185202.1:276** **----TAGGG..C...-GA......C...-----.A--GA....C..----..G.----TT**

**Platy-1-4b3_NW_012187201.1:328** **------GGG..C...-GA......C...-----.A--GA....C..----....----TT**

**Platy-1-4b3_NW_012190535.1:324** **--------G..C...-GA.A....C...-----.A--GA....C..----..G.----TT**

**Platy-1-5_NW_012163980.1:74925** **-----CAGG..CT.T-.........C..-----.A--G.....C..----.TG.----TT**

**Platy-1-5_NW_012164424.1:51747** **----TGAGGG.CT..-G........T.A-----.A--G.....C..----.TG.----TT**

**Platy-1-5_NW_012166868.1:18448** **-------GGG.C..T-G...........-----.A--G.....C..----.TG.----TC**

**Platy-1-5_NW_012167202.1:13968** **--------GG.C.T.-G........T..-----.A--G.....C..----....----TT**

**Platy-1-5_NW_012169090.1:46811** **--------GG.C...-G........T..-----.A--G.....C..----..G.----TT**

**Platy-1-5_NW_012173423.1:20889** **--------GG.C..T-G........T..-----.A--G..A..C..----..G.----TT**

**Platy-1-5_NW_012178979.1:21624** **-----------T.A.-G........T..-----.A--G.....C..----....----TT**

**Platy-1-5_NW_012183869.1:10206** **-------GGG.C.A.-G........T..-----.A--G.....C..----..G.----TT**

**Platy-1-5_NW_012184203.1:40477** **-------GGG.T..T-GA.......T..-----.A--G........----.TG.----TT**

**Platy-1-5_NW_012184979.1:15607** **-----TGGGG.C.A.-G........T..-----.A--G.....C..----....----TT**

**Platy-1-5_NW_012185158.1:92283** **------GGGG.C.T.-GA.......T..-----.A--G.....C..----....----TT**

**Platy-1-5_NW_012185880.1:91983** **-------GGG.C.T.-G........T..-----.A--G.....C..----..G.----TT**

**Platy-1-5_NW_012185945.1:596-6** **-------GG..CT.T-G...........-----.A--G.....C..----..G.----TT**

**Platy-1-5_NW_012187313.1:37890** **----TGGGG..CT..-G...........-----.A--G.....C..----....----TT**

**Platy-1-5_NW_012166980.1:44419** **----CAGGGG.CA..-G........T.T------GT-G.....CC.----.TG.----TT**

**Platy-1-5_NW_012167274.1:17-11** **------GGGG.CA..-G........T.T------GT-G.....CC.----.TG.----TT**

**Platy-1-5_NW_012169424.1:10725** **------GGG..CT..-G........T.T-----.A--G....TC..----..GT----TT**

**Platy-1-5_NW_012178219.1:204-3** **-----GGGG..CT..-............-----.A--G.....C..----..C.----TT**

70 80 90 100 110 120

....|....|....|....|....|....|....|....|....|....|....|....|

**Platy-1-1_NW_012163313.1:49684** **TAGGAGGC-----------TAGG-----CTAGCTGTTCA-AGG----CCAGCCT-GGGCA**

**Platy-1-1_NW_012163535.1:17840** **G......T-----------G...-----T...GC....G-...----.......-.....**

**Platy-1-1_NW_012162423.1:29766** **G.......-----------G...-----TC..GCC...C-...----.......-.....**

**Platy-1-1_NW_012163535.1:32779** **A.A.G...-----------A...-----..G.GA....G-..A----.......-...G.**

**Platy-1-1_NW_012163536.1:46396** **-.T...AT-----------G...-----....G......-...----.......-T....**

**Platy-1-1_NW_012169090.1:29360** **G.......-----------G...-----TC..GC.....-...----.......-.....**

**Platy-1-1_NW_012170313.1:34330** **A......T-----------G..T-----....GCA..TG-...----....G..-.A...**

**Platy-1-1_NW_012170424.1:19233** **A.......-----------A...-----.CG.G......-...----.......-.....**

**Platy-1-1_NW_012171090.1:58916** **A.......-----------A...-----....G......-...----.......-.....**

**Platy-1-1_NW_012171312.1:20970** **G.......---------------------...GC...TG-...----.......-.....**

**Platy-1-1_NW_012171312.1:22155** **A.......-----------G...-----GC..A......-..A----.......-...T.**

**Platy-1-1_NW_012172090.1:24950** **G.C....T-----------G...-----.C..GC...TGT..-----.......-.CA..**

**Platy-1-1_NW_012173534.1:35510** **A......T-----------G...-----TC..GC.....-...----.....A.-.....**

**Platy-1-1_NW_012175091.1:28244** **A....A.A-----------G...-----.C..GA.....-...----.......-.T...**

**Platy-1-1_NW_012176869.1:33712** **A.......-----------A...-----....G....TG-...----.......-.....**

**Platy-1-1_NW_012177091.1:48611** **G.......-----------G..------TC..GC.....-...----.......-.....**

**Platy-1-1_NW_012177646.1:34834** **G...CA..-----------G..A-----.C..G......-...----.......-.....**

**Platy-1-1_NW_012177979.1:10635** **A....A.T-----------G...-----TC..GCA...G-...----....T..-.AAA.**

**Platy-1-1_NW_012178646.1:26805** **G......G-----------A...-----TC..GCA...G-...----.......-.....**

**Platy-1-1_NW_012183958.1:14675** **G.......-----------A...-----...TGC...-TG...----.......-.....**

**Platy-1-1_NW_012184014.1:30269** **G.A...T.-----------A...-----TC..GC....G-...----.......-..A..**

**Platy-1-1_NW_012184214.1:36295** **G.......-----------A...-----T...GC....G-...----.......-...T.**

**Platy-1-1_NW_012184536.1:15341** **G.......-----------A..A-----T...G......-...----......-T.....**

**Platy-1-1_NW_012184613.1:35624** **G......A-----------G...-----TC..GC...TG-..A----.......-.....**

**Platy-1-1_NW_012184646.1:76762** **G......T-----------G..A-----....GCA...G-...----TT.....-...A.**

**Platy-1-1_NW_012184647.1:17260** **.......T-----------G...-----.C..GCA....-...----....T..-.....**

**Platy-1-1_NW_012184746.1:45925** **A..A...T-----------....-----T...GA....G-...----......C-.....**

**Platy-1-1_NW_012184979.1:12918** **A.......-----------A...-----....GCA....-G..----.-.....-.....**

**Platy-1-1_NW_012185580.1:14037** **G....A.T-----------G...-----....GCA....-...----.......-...A.**

**Platy-1-1_NW_012185646.1:80324** **G.A....T-----------G..A-----.C..GCA..TG-...----...A..CC.....**

**Platy-1-1_NW_012186090.1:22025** **A..AGA.T-----------G...-----.C..GA...TG-..A----.......-...T.**

**Platy-1-1_NW_012186313.1:66308** **G.......-----------G.A.-----.C..ACA..GG-..A----..T.TT.-.....**

**Platy-1-1_NW_012187646.1:68445** **A..A....-----------A...-----.C..GAA..TG-..A----.......-.....**

**Platy-1-1_NW_012187757.1:36641** **A.......-----------A...-----.C..AA..CTG-..A----.......-.....**

**Platy-1-1_NW_012187946.1:15918** **GT.....T-----------G...-----....GCA....-...----T......-..TT.**

**Platy-1-1_NW_012188868.1:11813** **A......T-----------G...-----.C..GC....G-...----T......-.....**

**Platy-1-1_NW_012189090.1:4979-** **GT......-----------G...-----....GA.....-...----..C....-AA...**

**Platy-1-1_NW_012189423.1:15745** **G....C.T-----------G..A-----TC..GCA....-...----.T.T...-.....**

**Platy-1-1_NW_012189423.1:49188** **A..A....-----------A...-----.A..GCT..TG-..A----.......-.T...**

**Platy-1-1_NW_012189424.1:43236** **G.......-----------A...-----TC..G....TG-...----.......-.....**

**Platy-1-1_NW_012190534.1:27184** **G......T-----------G...-----TC..GCA...G-...----....T.-T.....**

**Platy-1-1_NW_012190979.1:22614** **G.......-----------A...-----TC..GC...TG-...----.......-.....**

**Platy-1-2_NW_012164091.1:26806** **G......G-----------G...-----...CG....TG-..T----.......-.....**

**Platy-1-2_NW_012164091.1:26906** **G......G-----------G...-----...CG....TG-..T----.......-.....**

**Platy-1-2_NW_012165424.1:13538** **A.......-----------G...-----...CGC....G-...----....G..-.....**

**Platy-1-2_NW_012169090.1:16710** **G.......-----------G...-----...CGCA...G-...----.......-.....**

**Platy-1-2_NW_012169535.1:14903** **G....A..-----------A...-----...CG....-TG..T----.......-.....**

**Platy-1-2_NW_012172423.1:21747** **A.......-----------G...-----...CTCT....-..C----....T.-T.....**

**Platy-1-2_NW_012172535.1:90777** **G.......-----------A...-----...TGC....G-...----.......-.....**

**Platy-1-2_NW_012173534.1:79384** **G.......AAGGCTACGGCG...-----...CG.....G-.A.----.......-.....**

**Platy-1-2_NW_012176424.1:26496** **C....A..-----------G...-----..TCG....-TG...----.T.....-.....**

**Platy-1-2_NW_012184158.1:19413** **G.....AT-----------G...-----...TGC....G-...----.A..A..-.....**

**Platy-1-2_NW_012184202.1:16754** **G.......-----------A...-----...TGC....G-...----....G..-.....**

**Platy-1-2_NW_012184980.1:57576** **G.......-----------A...-----...TGC....G-..A----A......-.....**

**Platy-1-2_NW_012185302.1:16119** **G.......-----------G...-----...CA....-TG...----.-.T...-.....**

**Platy-1-2_NW_012187201.1:60133** **G.....AT-----------GT..-----...CAC....C-...----..T....-.....**

**Platy-1-2_NW_012187202.1:21081** **G..A....-----------G.A.-----...TGC.....-...----.......-.....**

**Platy-1-2_NW_012187325.1:43653** **G.......-----------GG..-----G..TGC.....-...----.......-.....**

**Platy-1-2_NW_012188646.1:21539** **G.......-----------A...-----...TAC.....-...----....A..-.....**

**Platy-1-2_NW_012189423.1:10665** **G......T-----------G...-----...TG....-CA...----.......-.....**

**Platy-1-2_NW_012189423.1:12584** **G......T-----------G...-----...TGC....C-...----..C....-.A...**

**Platy-1-2_NW_012189424.1:90770** **G......T-----------A...-----...CGCA...G-...----.......-.A...**

**Platy-1-2_NW_012189757.1:60581** **G.....AT-----------G...-----...TGCA...G-...----.G.....-.....**

**Platy-1-2_NW_012190868.1:93211** **G.......-----------A...-----...TGCAC...-.A.----A..A...-.....**

**Platy-1-2a_NW_012162647.1:5295** **G.....C.-----------G...-----...CG....-CG...----.......-.....**

**Platy-1-2a_NW_012163869.1:6833** **G---..TT-----------G...-----...CACA...G-...----.......-A....**

**Platy-1-2a_NW_012164202.1:3713** **G.......-----------G...-----...TGC....G-...----.......-.....**

**Platy-1-2a_NW_012164424.1:6158** **A......T-----------G...-----...CAC.C--.-...----.T.....-...T.**

**Platy-1-2a_NW_012165424.1:1861** **G...---T-----------G...-----...CG....-CG...----.T.....-.....**

**Platy-1-2a_NW_012167424.1:7138** **A..A....-----------G...-----...CGCA..TG-...----.......-.....**

**Platy-1-2a_NW_012167646.1:1017** **G.......-----------G...-----...TGCT..TG-...----.T..G..-...A.**

**Platy-1-2a_NW_012167868.1:8952** **A.......-----------A...-----...CA....-CG...----.......-.....**

**Platy-1-2a_NW_012170868.1:3906** **G.......-----------G...-----...CGCA..TG-...----.......-..ATG**

**Platy-1-2a_NW_012171312.1:1107** **G...----------------...-----...CG....-CG...----.......-.....**

**Platy-1-2a_NW_012172535.1:1491** **G......T-----------G...-----..GCACA...G-...----.......-.....**

**Platy-1-2a_NW_012173534.1:4251** **G.......-----------A...-----...CA....-CG...----...A...-.....**

**Platy-1-2a_NW_012173535.1:9675** **G.......-----------A...-----...TGC....G-...----.......-.....**

**Platy-1-2a_NW_012176424.1:1014** **G.......-----------G...-----...CGCA...G-...----T..A...-.....**

**Platy-1-2a_NW_012176868.1:1095** **A.......-----------G...-----...CAC....G-...----T....A.-.A...**

**Platy-1-2a_NW_012177979.1:9460** **G..A...T-----------G...-----...CACA..TG-...----...A...-CA..G**

**Platy-1-2a_NW_012178424.1:8756** **G......T-----------G...-----...TGC....G-...----....A..-.....**

**Platy-1-2a_NW_012179535.1:1101** **G......T-----------G...-----...CAC...-CA...----.T.....-A....**

**Platy-1-2a_NW_012183869.1:1597** **C.AT....-----------A...-----...TGC.....-...----A......-.....**

**Platy-1-2a_NW_012184025.1:1425** **GG...T..-----------G...-----...CGC...-CG...----.......-.....**

**Platy-1-2a_NW_012184091.1:5783** **G...CA..-----------G...-----...CGC...-CG...----.......-..T..**

**Platy-1-2a_NW_012184202.1:1281** **G.....TT-----------G..T-----..CCA....-CG..C----...A...-.....**

**Platy-1-2a_NW_012184358.1:8743** **G....T.T-----------G...-----..CCG....-CG...----.......-.....**

**Platy-1-2a_NW_012184757.1:4571** **G......T-----------G...-----...TGC...TG-.AA----.......-.....**

**Platy-1-2a_NW_012184802.1:5276** **G.......-----------C...-----...CG....-CG...----.......-.....**

**Platy-1-2a_NW_012184857.1:1202** **G.A.....-----------G...-----...CG....-CG...----...A...-.....**

**Platy-1-2a_NW_012184868.1:5356** **G.......-----------A...-----...CGC...TG-...----.......-.....**

**Platy-1-2a_NW_012185025.1:8158** **G.......-----------A...-----...CA....-CG...----.T.....-.....**

**Platy-1-2a_NW_012185313.1:4572** **G.......-----------G...-----...TGC...-CG...----..TA...-.....**

**Platy-1-2a_NW_012186868.1:3979** **G.A.T..T-----------G...-----...CG....-CG...----.....A.-...A.**

**Platy-1-2a_NW_012187269.1:8608** **G.......-----------A...-----...TGCA...G-.A.----....G..-.....**

**Platy-1-2a_NW_012187424.1:6175** **G..A....-----------A...-----...CAC...TG-...----.......-.....**

**Platy-1-2a_NW_012188312.1:2203** **G...G...-----------A...-----...CGC....G-...----.......-.....**

**Platy-1-2a_NW_012188646.1:2664** **G.......-----------G...-----...TGC....G-...----......G-.....**

**Platy-1-2a_NW_012189312.1:9461** **AG.....T-----------G...-----...CACAC--.-...----.T.....-...T.**

**Platy-1-2a_NW_012189423.1:1252** **G......T-----------G...-----...TTC....G-...----.......-...A.**

**Platy-1-2a_NW_012191312.1:2658** **GG......-----------A...-----...CGC....G-...----..C....-.....**

**Platy-1-2b_NW_012162425.1:6193** **A......T-----------....-----....GA.....-.AA----.......-.....**

**Platy-1-2b_NW_012165757.1:1879** **A......T-----------GG..-----T..CACA...G-...----.......-....G**

**Platy-1-2b_NW_012165757.1:2715** **G.......-----------G...-----..GTG....-CA...----.......-.....**

**Platy-1-2b_NW_012165980.1:1672** **G......T-----------G...-----...TGA....G-...----.......-.....**

**Platy-1-2b_NW_012166091.1:5131** **G.......-----------G...-----...CG......-...----.......-.....**

**Platy-1-2b_NW_012166313.1:3251** **A......A-----------G...-----T..CGA.....-...----.......-.....**

**Platy-1-2b_NW_012166535.1:5728** **G.......-----------G...-----...CACA...G-...----.......-...A.**

**Platy-1-2b_NW_012166868.1:4843** **G.......-----------G...-----...TGC...-.G...----.TG....-...T.**

**Platy-1-2b_NW_012167202.1:5072** **G....T..-----------G...-----...CA......-...----...A...-.....**

**Platy-1-2b_NW_012167313.1:2947** **G.....C.-----------G...-----...CAC...TG-...----.T.....-..A..**

**Platy-1-2b_NW_012169313.1:3109** **G......T-----------G...-----...CGCA....-...----......-T.....**

**Platy-1-2b_NW_012172201.1:9584** **C.......-----------A...-----...CA....-CG...----.......-..T..**

**Platy-1-2b_NW_012173534.1:2304** **G......T-----------G...-----...TGCA...G-...----......CT.....**

**Platy-1-2b_NW_012173534.1:2643** **A.......-----------G...-----.C.CG...CAG-..A----.......-..A..**

**Platy-1-2b_NW_012173535.1:2973** **A.......-----------A...-----...CAG.....-G..----.......-.....**

**Platy-1-2b_NW_012174646.1:7972** **G....A..-----------A...-----...CA....-CG...----....T..-.....**

**Platy-1-2b_NW_012175796.1:638-** **A.......-----------G...-----.C.CG...CAG-..A----.......-..A..**

**Platy-1-2b_NW_012180543.1:1009** **G......T-----------G.A.-----...TGC....G-...----.......-.....**

**Platy-1-2b_NW_012180868.1:6439** **G.......-----------G...-----...TGCA.G.C-...----.......-.....**

**Platy-1-2b_NW_012181646.1:7893** **G.......-----------G...-----...CA....-CG...----.......-.....**

**Platy-1-2b_NW_012182423.1:7347** **G.....A.-----------G...-----...TGC...-CG...----.......-.....**

**Platy-1-2b_NW_012183534.1:1116** **A..T....-----------A.A.-----T..CAA.....-...----.......-.A.T.**

**Platy-1-2b_NW_012183534.1:1895** **G.......-----------A..A-----..--GCA...G-...----....T.-T.....**

**Platy-1-2b_NW_012183868.1:7418** **G.......-----------G...-----...CG......-...----..C....-.....**

**Platy-1-2b_NW_012183869.1:1188** **G..T....-----------G...-----A..CGC...TG-...----...A...-.A...**

**Platy-1-2b_NW_012184424.1:3175** **G.......-----------G..C-----...CGC.....-...----.......-...AT**

**Platy-1-2b_NW_012185114.1:1027** **GC.....T-----------A...-----...CACA...T-...----......-T.....**

**Platy-1-2b_NW_012186090.1:1197** **G......---------------------...CGC....C-...----.T....C-.....**

**Platy-1-2b_NW_012186202.1:4485** **A.......-----------A..A-----...TGC....G-.A.----.......-.....**

**Platy-1-2b_NW_012186313.1:7737** **G.A.....-----------A...-----...CAC...-CG...----.....G.-...A.**

**Platy-1-2b_NW_012186424.1:3482** **A.....AT-----------G...-----...CACA...TG...----....T..-.....**

**Platy-1-2b_NW_012187424.1:5967** **G.......-----------A...-----...TGCA..-CA...----..G....-.....**

**Platy-1-2b_NW_012188090.1:1455** **G..T...T-----------G...-----...TG....-CG...----.......-..T..**

**Platy-1-2b_NW_012188090.1:1622** **G.......-----------A...-----....G......-..A----.......-....C**

**Platy-1-2b_NW_012188646.1:1667** **G.......-----------G..A-----...CGCA.GTG-...----....T.CT.....**

**Platy-1-2b_NW_012188757.1:8687** **G.A.....-----------A...-----.C.CACA..-TG...----.......-.....**

**Platy-1-2b_NW_012189090.1:5283** **G......T-----------G..A-----...CGC...-TA..C----T......-.....**

**Platy-1-2b_NW_012189423.1:1292** **........-----------A.A.-----...TG....-CG...----.......-.....**

**Platy-1-2b_NW_012189757.1:6380** **G.......-----------A...-----...TG....-GG...----.......-.....**

**Platy-1-2b_NW_012190534.1:2742** **A.......-----------G.T.-----T.GCAC..C.G-...----.......-.....**

**Platy-1-2b_NW_012190646.1:3121** **G....A.T-----------G..A-----...CG.....G-.T.----.......-...A.**

**Platy-1-2b_NW_012191312.1:2401** **G......T-----------G...-----G..CAC..A.G-...----.......-.....**

**Platy-1-3_NW_012162423.1:14651** **G.A.....-----------G..A-----...TG....TG-...----.......-.....**

**Platy-1-3_NW_012163091.1:14454** **G......T-----------G...-----...TGCA..TG-...----...A...-...A.**

**Platy-1-3_NW_012163202.1:30198** **A......T-----------G...-----...CGA...-TG...----.......-.....**

**Platy-1-3_NW_012164869.1:60849** **G.......-----------G...-----...TG.A..TG-...----...A...-.....**

**Platy-1-3_NW_012165198.1:5928-** **G.A.....-----------G...-----...TGCA..TG-...----.......-..T..**

**Platy-1-3_NW_012165535.1:14696** **G.......-----------G...-----...TG..C.-TG...----...AT..-.....**

**Platy-1-3_NW_012167535.1:56481** **G.A....T-----------G...-----...TGCA..TG-...----..CAT..-.....**

**Platy-1-3_NW_012170313.1:12544** **G....A.T-----------G...-----...TG.T..T.-...----T......-.....**

**Platy-1-3_NW_012170535.1:46745** **G......T-----------G...-----...TGCA..AG-...----....A..-.....**

**Platy-1-3_NW_012173534.1:14650** **G...G..T-----------G...-----...TGCA...G-...----..T....-.....**

**Platy-1-3_NW_012173534.1:34177** **G.......-----------A.A.-----...AG....TG-...----.......-.....**

**Platy-1-3_NW_012174202.1:58320** **G.......-----------G...-----...TG.A..-CG...----.......-.....**

**Platy-1-3_NW_012174424.1:28994** **A.A....T-----------G...-----..CTG....-CG...----.......-.....**

**Platy-1-3_NW_012178646.1:42536** **G.......-----------G...-----..GTGCA..TG-...----.......-.....**

**Platy-1-3_NW_012179646.1:13674** **A---G..T-----------G...-----....G....TG-...----.......-.....**

**Platy-1-3_NW_012179646.1:13765** **A---G..T-----------G...-----....G....TG-...----.......-.....**

**Platy-1-3_NW_012180757.1:22137** **G...G...-----------A...-----G..TG....TG-...----.......-.....**

**Platy-1-3_NW_012181868.1:10555** **G......T-----------G...-----...TGC-------------...A...-.....**

**Platy-1-3_NW_012183892.1:21329** **G..A....-----------G...-----T..CA....-TG...----...T...-.....**

**Platy-1-3_NW_012184003.1:21311** **G......T-----------G...-----...TG....TG-...----.......-..A..**

**Platy-1-3_NW_012184092.1:29931** **G......T-----------G...-----T.GCA....-TG...----.T.T...-.....**

**Platy-1-3_NW_012184092.1:47861** **G.......-----------A...-----...TA....-TG.T.----.......-...G.**

**Platy-1-3_NW_012185313.1:81785** **A.....AT-----------G..--------.CG.A..-TG.T.----.......-...T.**

**Platy-1-3_NW_012185535.1:12734** **G.......-----------A...-----...TGCA..-TG.A.----.T.....-.....**

**Platy-1-3_NW_012185757.1:66791** **G......T-----------G...-----...TG......-...----.T.....-.....**

**Platy-1-3_NW_012186720.1:3413-** **G.......-----------A..A-----..GTGCA..TG-...----.......-C....**

**Platy-1-3_NW_012186757.1:60120** **G..A...T-----------G...-----....G....T.-...----A......-.....**

**Platy-1-3_NW_012188090.1:38232** **G.....AT-----------G...-----..GTGG...TG-...----.......-..A..**

**Platy-1-3_NW_012188458.1:41468** **G...G...-----------A...-----...TGCA..------------.....-...TG**

**Platy-1-3_NW_012191423.1:67979** **G....A..-----------G...-----..GTG.T.C-G-...----T......-.....**

**Platy-1-4_NW_012173980.1:20137** **G..-...T-----------G...-----...TGA.....T...----...A...-.....**

**Platy-1-4_NW_012189423.1:11596** **G.......-----------G...-----...TGA.....-...----...AA..-.....**

**Platy-1-4_NW_012189868.1:48523** **G.......-----------G...-----...TAA.....-...----...A...-....T**

**Platy-1-4_NW_012190201.1:56706** **G......T-----------G...-----...TGA.....-...----.......-.....**

**Platy-1-4a_NW_012162647.1:5343** **G...---.-----------G...-----...TGC.....-...----...A...-.....**

**Platy-1-4a_NW_012162869.1:5364** **G.......-----------G...-----...TGC.....-...----...A...-C....**

**Platy-1-4a_NW_012162869.1:5365** **G.......-----------G...-----...TGC.....-...----...A...-.....**

**Platy-1-4a_NW_012163158.1:1929** **G.......-----------G...-----..GTGC.....-...----...A...-.....**

**Platy-1-4a_NW_012163535.1:2382** **G.......-----------G...-----...TGC.....-...----...A...-.....**

**Platy-1-4a_NW_012163980.1:1247** **G.......-----------G...-----...TGC.....-...----...A...-.....**

**Platy-1-4a_NW_012164202.1:2548** **G......T-----------G...-----...TGC.....-...----...A...-.....**

**Platy-1-4a_NW_012164424.1:2374** **G.......-----------G...-----...TGC.....-...----...A...-.....**

**Platy-1-4a_NW_012164646.1:1456** **G......T-----------G...-----...TGC-------------...A...-.....**

**Platy-1-4a_NW_012165091.1:1698** **G.......-----------A...-----...TG......-...----...AT..-.....**

**Platy-1-4a_NW_012165091.1:2771** **G......T-----------G...-----...TGC.....-...----...A...-.....**

**Platy-1-4a_NW_012165091.1:5564** **G.......-----------G...-----...TGC.....-...----...A...-.....**

**Platy-1-4a_NW_012165535.1:1152** **G.......-----------G...-----...TGC.....-...----...A...-.....**

**Platy-1-4a_NW_012165757.1:4481** **G.......-----------G...TTATG...TGC.....-...----...A...-.....**

**Platy-1-4a_NW_012165757.1:4482** **G.......-----------G...CTATG...TGC.....-...----...A...-.....**

**Platy-1-4a_NW_012165757.1:8542** **A.......-----------A...-----...TGC.....-...--------------...**

**Platy-1-4a_NW_012165980.1:3779** **G.......-----------G...-----...TG......-...----...A...-.....**

**Platy-1-4a_NW_012166202.1:2027** **G...---.-----------A...-----...TGC.....-...----...AT..-.....**

**Platy-1-4a_NW_012166646.1:3729** **G.......-----------G...-----A..TGCA.C..-...----...A...-.....**

**Platy-1-4a_NW_012167202.1:4991** **G.......-----------G...-----...TGCA....-...----...AG..-.....**

**Platy-1-4a_NW_012167313.1:3173** **G..T....-----------G...-----...TG......-.C.----...A...-.....**

**Platy-1-4a_NW_012167424.1:2226** **G.......-----------G...-----...TGC.....-...----...AG..-.....**

**Platy-1-4a_NW_012167868.1:6778** **G.......-----------G...-----...TGC.....-...----...A...-....G**

**Platy-1-4a_NW_012168313.1:1440** **G.......-----------G...-----..TTGC.....-..A----...A...-....C**

**Platy-1-4a_NW_012168424.1:4326** **G..--------------------------------------------T..A...-.....**

**Platy-1-4a_NW_012168646.1:1504** **G.......-----------G...-----...TGC.....-...----...A...-..A..**

**Platy-1-4a_NW_012168979.1:8062** **G.......-----------G...-----...TGC.....-...----...A...-.....**

**Platy-1-4a_NW_012169090.1:2106** **G.......-----------G...-----...TGCA....-...----...A...-.....**

**Platy-1-4a_NW_012169090.1:4300** **G.......-----------G...-----..GTG......-...----...A...-.....**

**Platy-1-4a_NW_012169091.1:2859** **G......T-----------G.A.-----...TGC.....-...----...A...-.....**

**Platy-1-4a_NW_012169313.1:2654** **G.......-----------G...-----...TGC.....-...----.A.A.T.-.....**

**Platy-1-4a_NW_012169535.1:7280** **G.......-----------G...-----...TGC.....-...----T..AG..-.....**

**Platy-1-4a_NW_012169979.1:4186** **G......----------------------------------------...A.T.-.....**

**Platy-1-4a_NW_012170201.1:1053** **G.---...-----------A...-----...TGC.....-...----...A...-.....**

**Platy-1-4a_NW_012171126.1:2521** **G......T-----------G...-----...TGC.....-...----...A...-.....**

**Platy-1-4a_NW_012171201.1:1320** **A.......-----------G...-----...TGC.....-...----...A...-.....**

**Platy-1-4a_NW_012171312.1:1877** **G.......-----------A...-----...TGC-C.T.-...----...A...-.....**

**Platy-1-4a_NW_012171312.1:5539** **G...C...-----------G.A.-----...TGC.....-...----...AT..-.....**

**Platy-1-4a_NW_012173201.1:4719** **G....A.T-----------G...-----...TGC.....-...----...A...-..-..**

**Platy-1-4a_NW_012173312.1:2571** **G......T-----------G...-----...TGCA....-...----.T.A...-.....**

**Platy-1-4a_NW_012173758.1:3202** **G.......-----------A..A-----...TGC.....-...----...A...-.....**

**Platy-1-4a_NW_012173758.1:3282** **G.......-----------G.A.-----...TGC.....-...----...A...-....G**

**Platy-1-4a_NW_012174424.1:7477** **G.......-----------G...-----...TGC.....-...----...A...-.....**

**Platy-1-4a_NW_012174646.1:1101** **G.......-----------A...-----...TGC.....-...----...A...-.....**

**Platy-1-4a_NW_012175646.1:4412** **G..A....-----------G...-----...TG......-...----...AG..-..C..**

**Platy-1-4a_NW_012176202.1:1353** **G.......-----------G...-----...TGC.....-...----...A...-.....**

**Platy-1-4a_NW_012176313.1:3041** **G.......-----------G...-----...TGC.....-...----...AT..-.....**

**Platy-1-4a_NW_012176535.1:1424** **G.......-----------G...-----...TGC.....-...----...A...-.....**

**Platy-1-4a_NW_012176646.1:1884** **G.......-----------G...-----...TGC.....-...----...A...-.....**

**Platy-1-4a_NW_012176757.1:2674** **G.......-----------A...-----...TG......-...----...A...-.....**

**Platy-1-4a_NW_012177979.1:2731** **G......T-----------G...-----G..TG...C..-...----.....T.-.....**

**Platy-1-4a_NW_012178025.1:1167** **G.A.....-----------G...-----...TGCA....-...----...AG..-.....**

**Platy-1-4a_NW_012178091.1:1955** **G......T-----------G...-----...TGC.....-...----...A...-.....**

**Platy-1-4a_NW_012178535.1:1006** **G.......-----------G...-----...TGC.....-...----...A...-.....**

**Platy-1-4a_NW_012178757.1:4212** **G......T-----------G...-----...TGCA....-...----...A...-.....**

**Platy-1-4a_NW_012181312.1:9214** **G.......-----------G...-----...TG......-...----...AG..-.....**

**Platy-1-4a_NW_012182535.1:1154** **G.......-----------A...-----...TGC.....-..A----...A...-.....**

**Platy-1-4a_NW_012183201.1:3224** **G.......-----------G...-----...TGC.....-...----...A...-.....**

**Platy-1-4a_NW_012183802.1:7811** **A.A.....-----------G...-----...TGC.....-...----...A...-.....**

**Platy-1-4a_NW_012183813.1:5526** **G.......-----------G...-----...TGC.....-...----...A...-.....**

**Platy-1-4a_NW_012183824.1:1101** **G.......-----------G...-----...TGC.....-...----...A...-.....**

**Platy-1-4a_NW_012183869.1:1041** **G.......-----------G...-----...TGC.....-...----...A...-.....**

**Platy-1-4a_NW_012183869.1:1360** **G.......-----------G...-----...TGC.....-...----...A...-.....**

**Platy-1-4a_NW_012183869.1:5673** **G.......-----------G...-----...TG......-...----...A...-.....**

**Platy-1-4a_NW_012183881.1:1201** **G.......-----------A...-----...TGC.....-.A.----...A...-.....**

**Platy-1-4a_NW_012183958.1:1156** **G.......-----------A...-----T..TGC.....-...----...A...-.....**

**Platy-1-4a_NW_012183980.1:1098** **G..A...T-----------G...-----...TG......-...----...A...-.....**

**Platy-1-4a_NW_012184047.1:9856** **G......T-----------C...-----...TGCC....-...----...A...-.....**

**Platy-1-4a_NW_012184147.1:3563** **G.......-----------A...-----...TGC.....-...----...A...-.....**

**Platy-1-4a_NW_012184269.1:1219** **G.......-----------G...-----...TGC.....-...----...AG..-.....**

**Platy-1-4a_NW_012184480.1:8638** **G.......-----------G...-----...TGC.....-...----...AT..-.....**

**Platy-1-4a_NW_012184535.1:3014** **G.......-----------G...-----...TG.T....-G..----...A...-.....**

**Platy-1-4a_NW_012184535.1:7443** **G.......-----------G...-----.AGTG......-...----...A...-.....**

**Platy-1-4a_NW_012184602.1:1032** **G.......-----------G.A.-----G..TGC.....-...----...AG..-.....**

**Platy-1-4a_NW_012184702.1:2422** **G.T.....-----------G...-----...TGCA....-...----...A...-.....**

**Platy-1-4a_NW_012184735.1:1484** **G.......-----------G...-----...TGC.....-...----...A...-.....**

**Platy-1-4a_NW_012184902.1:5722** **G..T...T-----------G...-----...TGC.....-...----...A...-.....**

**Platy-1-4a_NW_012184980.1:7322** **G.......-----------G...-----...TGC.....-...----...A...-.....**

**Platy-1-4a_NW_012185202.1:8626** **G.......-----------G...-----...TGC.....-...----...A...-.....**

**Platy-1-4a_NW_012185247.1:6115** **G.......-----------A...-----...TGC.....-...----...T...-.....**

**Platy-1-4a_NW_012185313.1:1077** **G.......-----------G...-----...TGCC...G-...----...A...-.....**

**Platy-1-4a_NW_012185313.1:9239** **G......T-----------G...-----...TGC.....-...----...AG..-.....**

**Platy-1-4a_NW_012185424.1:2354** **A......T-----------A...-----...TGCA....-...----...A...-.....**

**Platy-1-4a_NW_012185425.1:7570** **G......T-----------G...-----...TGC.....-...----...A...-.....**

**Platy-1-4a_NW_012185458.1:2034** **G.......-----------A.A.-----...TGC.....-...----G..A...-.....**

**Platy-1-4a_NW_012185757.1:1154** **G.......-----------G...-----...TGC.....-...----...A...-.....**

**Platy-1-4a_NW_012185757.1:1242** **G.......-----------G...-----...TGC.....-...----...A...-.....**

**Platy-1-4a_NW_012185913.1:1882** **G..A....-----------G...-----...TGC.....-...----...A...-.....**

**Platy-1-4a_NW_012185957.1:9479** **G.......-----------G...-----...TGC.....-.A.----...A...-.....**

**Platy-1-4a_NW_012186091.1:6351** **G.......-----------G...-----...TGC.....-...----...AG..-.....**

**Platy-1-4a_NW_012186114.1:2462** **G.......-----------A...-----T..TGC.....-...----...A...-.....**

**Platy-1-4a_NW_012186313.1:4701** **G.......-----------G...-----...TG......-...----...A...-.....**

**Platy-1-4a_NW_012186535.1:5683** **A..A....-----------G...-----...TGC.....-.C.----T..A...-.....**

**Platy-1-4a_NW_012186646.1:6551** **G.......-----------G...-----...TGCA....-...----...A...-.....**

**Platy-1-4a_NW_012186757.1:1166** **G.......-----------G...-----...TGC.....-...----...A...-.....**

**Platy-1-4a_NW_012186757.1:1875** **G....CA.-----------G...-----...TG......-...----...A...-.....**

**Platy-1-4a_NW_012186979.1:9295** **G.......-----------G...-----...TGC.....-...----...A...-.....**

**Platy-1-4a_NW_012187202.1:5070** **G.....T.-----------A...-----...TG......-...----...A...-.....**

**Platy-1-4a_NW_012187757.1:6197** **G.......-----------G...-----...TGC.....-...----...A...-.....**

**Platy-1-4a_NW_012188090.1:6532** **G.......-----------G...-----...TGC.....-...----...A...-.....**

**Platy-1-4a_NW_012188868.1:4269** **G.......-----------G...-----...TGC.....-...----...A.T.-.....**

**Platy-1-4a_NW_012189201.1:1245** **G.......-----------G...-----...TGC.....-...----...AG..-.....**

**Platy-1-4a_NW_012189201.1:2745** **G.......-----------A...-----...TGCA....-...----...A...-.....**

**Platy-1-4a_NW_012189423.1:1301** **G......T-----------G...-----...TG..C.G.-...----...A...-.....**

**Platy-1-4a_NW_012189423.1:2606** **G.......-----------G...-----...TGC.....-...----...AG..-T....**

**Platy-1-4a_NW_012189424.1:1588** **G.......-----------G...-----...TGCA....-...----...A...-.....**

**Platy-1-4a_NW_012189979.1:1974** **G.......-----------G...-----...TGC.....-...----...AG..-.....**

**Platy-1-4a_NW_012189979.1:3723** **G.......-----------G...-----...TACA....-...----...A...-.....**

**Platy-1-4a_NW_012190534.1:1765** **G.......-----------G...-----...TGC.....-...----...A...-.....**

**Platy-1-4a_NW_012190757.1:6050** **G.......-----------G..A-----...TGC.....-...----...A...-.....**

**Platy-1-4a_NW_012190868.1:3122** **G.....A.-----------G...-----...TGC.....-...----...AA..-.....**

**Platy-1-4a_NW_012191090.1:1473** **G...G...-----------G...-----...TGC.....-...----...AG..-.....**

**Platy-1-4a_NW_012191090.1:7220** **G.......-----------G...-----...TGC.....-...----...A..C-TT...**

**Platy-1-4a_NW_012191201.1:4096** **G.......-----------G...-----...TACA....-...----...A...-.....**

**Platy-1-4a_NW_012191423.1:5463** **G.......-----------G...-----...TGCA....-...----...A..C-.....**

**Platy-1-4b_NW_012162758.1:2572** **GG......-----------G...-----...TGA.....-GT.----...AG..-.....**

**Platy-1-4b_NW_012162869.1:6381** **G.......-----------G...-----...TGA.....-...----...AG..-.....**

**Platy-1-4b_NW_012163202.1:1994** **G.......-----------G...-----...TGA.....-..T----...C...-T...T**

**Platy-1-4b_NW_012163535.1:2530** **G.......-----------G...-----...TGA.....-.T.----...AG..-.....**

**Platy-1-4b_NW_012163535.1:2531** **G.......-----------G...-----...TGA.....-.T.----...AG..-.....**

**Platy-1-4b_NW_012163535.1:8297** **G.......-----------G...-----...TGA.....-.T.----...AG..-.....**

**Platy-1-4b_NW_012163647.1:6366** **G.......-----------G...-----...TGA...T.-...----...AG..-C....**

**Platy-1-4b_NW_012163926.1:5965** **G.......-----------G...-----...TGA.....-...----...AG..-.....**

**Platy-1-4b_NW_012164646.1:9630** **G.......-----------G...-----...TGA.....-.T.----...AG..-.....**

**Platy-1-4b_NW_012165757.1:5703** **G.......-----------G...-----...TGA.....-.T.----...AG..-..A..**

**Platy-1-4b_NW_012166868.1:1567** **G.......-----------A...-----...TGA.....-.T.----...AG..-.....**

**Platy-1-4b_NW_012167979.1:1849** **G.......-----------G...-----...TGA.....-.T.----...AG..-.....**

**Platy-1-4b_NW_012168979.1:4354** **G.......-----------G...-----...TGA.....-.T.----T..AG.C-T....**

**Platy-1-4b_NW_012169202.1:2313** **G......T-----------G...-----...TGA.....-...----...AG..-...T.**

**Platy-1-4b_NW_012169202.1:2314** **G......T-----------G...-----...TGA.....-...----...AG..-...T.**

**Platy-1-4b_NW_012169313.1:3048** **G.......-----------G...-----...TGA.....-.T.----...AG..-.....**

**Platy-1-4b_NW_012172423.1:1435** **G.......-----------G...-----...TGA.....-...----...AG..-.....**

**Platy-1-4b_NW_012172846.1:88-1** **A.......-----------G...-----...TGA.....-...----...AG..-.....**

**Platy-1-4b_NW_012173423.1:6003** **G.A.....-----------G...-----...TGA...G.-.T.----..TAG..-.....**

**Platy-1-4b_NW_012173534.1:2605** **G.......-----------G...-----...TGA.....-.T.----...AG..-.....**

**Platy-1-4b_NW_012173536.1:2838** **G.......GAGGCGAGGC-G...-----...TGA.....-..A----...AG..-.....**

**Platy-1-4b_NW_012174202.1:6446** **G.......-----------A...-----...TGC.....-.T.----...AG..-.....**

**Platy-1-4b_NW_012175568.1:1042** **G.......-----------G...-----...TGA....T-.T.----...AG..-.....**

**Platy-1-4b_NW_012179090.1:4388** **G.......-----------G...-----...TGA.....-.T.----...AG..-.....**

**Platy-1-4b_NW_012179424.1:2256** **G.A.....-----------G...-----...TGA.....-.T.----...AG..-.....**

**Platy-1-4b_NW_012180424.1:1829** **G.......-----------G...-----...TGA.....-.T.----...AG..-.....**

**Platy-1-4b_NW_012181312.1:4593** **G......T-----------G...-----...TGA.....-.T.----...AG..-.....**

**Platy-1-4b_NW_012181868.1:3187** **........-----------G...-----...TGA.....-.T.----...AG..-.....**

**Platy-1-4b_NW_012182423.1:1093** **G.......-----------G...-----...TGA.....-.T.----...AG..-.....**

**Platy-1-4b_NW_012182979.1:1332** **G.......-----------G...-----...TGA.....-.T.----...AG..-.....**

**Platy-1-4b_NW_012184003.1:1762** **G.......-----------G...-----...TGA....G-.T.----...AG..-.....**

**Platy-1-4b_NW_012184313.1:9985** **G.......-----------G...-----...TGA.....-.T.----...AG..-.....**

**Platy-1-4b_NW_012184436.1:2031** **G.......-----------G...-----...TGAA....-GT.----...AG..-.....**

**Platy-1-4b_NW_012184513.1:8005** **G.......-----------A...-----...TGA.....-.C.----...AG..-.....**

**Platy-1-4b_NW_012184646.1:1081** **G.......-----------A...-----...TGA.....-...----...AG..-.....**

**Platy-1-4b_NW_012184868.1:5660** **G.......-----------G...-----...TGG.....-...----...AG..-.....**

**Platy-1-4b_NW_012185091.1:4580** **G.......-----------G...-----...TGA.....-.T.----...AGA.-.....**

**Platy-1-4b_NW_012185202.1:3769** **G.......-----------G...-----...TGA.....-.T.----...AG..-.....**

**Platy-1-4b_NW_012185424.1:3710** **G.......-----------G...-----...TGA.....-...----...AG..-.....**

**Platy-1-4b_NW_012185424.1:3916** **G......T-----------G...-----...TGA....G-.T.----...AG..-.....**

**Platy-1-4b_NW_012185646.1:1492** **G......T-----------G...-----...TGA.....-.T.----...AG..-.....**

**Platy-1-4b_NW_012185757.1:1106** **G.......-----------G...-----...TGA....G-.T.----...AG..-.....**

**Platy-1-4b_NW_012185868.1:3666** **G.......-----------G...-----...TGA.....-...----...C...-...GG**

**Platy-1-4b_NW_012186091.1:1130** **G.......-----------G...-----...TGA.....-...----...AG..-.....**

**Platy-1-4b_NW_012186202.1:1115** **G.......-----------G...-----...CGA.....-.C.----...AG..-.....**

**Platy-1-4b_NW_012186535.1:9707** **G.......-----------G...-----...TGA.....-...----...AG..-.....**

**Platy-1-4b_NW_012186979.1:1006** **G.......-----------G...-----...TGA.....-.T.----...AG..-.....**

**Platy-1-4b_NW_012187313.1:1001** **G.....A.-----------G...-----...TGA.....-.T.----...AG..-.....**

**Platy-1-4b_NW_012188312.1:6490** **G.......-----------G...-----...TGA....G-.T.----...AG..-.....**

**Platy-1-4b_NW_012188480.1:1196** **A.......-----------G...-----...TGA.....-.T.----...AG.A-.....**

**Platy-1-4b_NW_012189423.1:1089** **G......T-----------G...-----...CGA.....-.T.----...AG..-.....**

**Platy-1-4b_NW_012189423.1:1825** **G......T-----------G...-----...TGA.....-...----...AG..-.....**

**Platy-1-4b_NW_012189423.1:1887** **G.......-----------G...-----...TGA...TG-.T.----...AG..-.....**

**Platy-1-4b_NW_012190080.1:706-** **G.......-----------G...-----...TGA.....-...----...AG..-.....**

**Platy-1-4b_NW_012190534.1:9595** **G......T-----------G...-----...TGA.....-.T.----...AG..-.....**

**Platy-1-4b_NW_012190868.1:1499** **G.......-----------G...-----...TGA.....-.T.----...AG..-.....**

**Platy-1-4b_NW_012191201.1:6367** **G.......-----------G...-----...TGA.....-GT.----...AG..-.....**

**Platy-1-4b_NW_012191312.1:2418** **G.......-----------G...-----...TGA.....-.T.----...AG.A-.....**

**Platy-1-4b3_NW_012172535.1:174** **G.......-----------G...-----...TGA....G-.T.----...AG..-.....**

**Platy-1-4b3_NW_012180979.1:316** **G.......-----------G...-----...TGA....G-.T.----...AGT.-.....**

**Platy-1-4b3_NW_012181312.1:304** **G.......-----------G...-----...TGA....G-.T.----...AG..-.....**

**Platy-1-4b3_NW_012184424.1:305** **G.......-----------G...-----...TGA....G-.T.----...AG..-.....**

**Platy-1-4b3_NW_012184591.1:794** **G.......-----------G...-----...TGA....G-.T.----...AG..-.....**

**Platy-1-4b3_NW_012184868.1:557** **G.......-----------G...-----...TGA.....-.T.----...AG..-.....**

**Platy-1-4b3_NW_012185158.1:100** **G.......-----------A...-----...TGA....G-.T.----...AG..-.....**

**Platy-1-4b3_NW_012185202.1:276** **G.......-----------G...-----...TGA....G-.T.----...AGG.-.....**

**Platy-1-4b3_NW_012187201.1:328** **G.......-----------G...-----...TGA....G-.T.----...CG..-.....**

**Platy-1-4b3_NW_012190535.1:324** **G.......-----------G.C.-----...TGA....G-.T.----...A...-.....**

**Platy-1-5_NW_012163980.1:74925** **G......T-----------G...-----...TGC.....-...----.......-.....**

**Platy-1-5_NW_012164424.1:51747** **G.....CT-----------G...-----...TGC...-CG...CCAA...A...-.....**

**Platy-1-5_NW_012166868.1:18448** **G.....C.-----------A...-----..GTGC...-CG...CCAA...A...-.....**

**Platy-1-5_NW_012167202.1:13968** **A.....T.-----------G...-----...TG.A..TCG...TCAA...A...-.....**

**Platy-1-5_NW_012169090.1:46811** **G.....CT-----------G...-----...TGCA..-CG...CCAA...A...-.....**

**Platy-1-5_NW_012173423.1:20889** **G......T-----------G...-----...TG....-CGG..CCAA----...-.....**

**Platy-1-5_NW_012178979.1:21624** **G.....C.-----------G...-----...TG....-TG...CCAA...A...-.....**

**Platy-1-5_NW_012183869.1:10206** **G.......-----------G...-----..CTGC...-CG...CCAA...A...-.....**

**Platy-1-5_NW_012184203.1:40477** **G.......-----------G...-----...TGCA..-TG...----....T..-.....**

**Platy-1-5_NW_012184979.1:15607** **G......T-----------G...-----...TGC...-CG...----...A...-.....**

**Platy-1-5_NW_012185158.1:92283** **A......T-----------G...-----...TGCA..-CG...----...A...-.....**

**Platy-1-5_NW_012185880.1:91983** **G.......-----------G...-----...TGC...-GG...CCAA...A..C-.....**

**Platy-1-5_NW_012185945.1:596-6** **C.......-----------G...-----...TGC....G-...----...A...-..A..**

**Platy-1-5_NW_012187313.1:37890** **G.......-----------G...-----...TA....TG-...----...A..A-.A...**

**Platy-1-5_NW_012166980.1:44419** **G.......-----------G...-----...CGC...-CC...----....T..-.....**

**Platy-1-5_NW_012167274.1:17-11** **G.......-----------G...-----...CGC...-CC...----....T..-.....**

**Platy-1-5_NW_012169424.1:10725** **G......A-----------G...-----....GC...-CG...----....T.C-..T..**

**Platy-1-5_NW_012178219.1:204-3** **G.......-----------G...-----...TGC....G-...----...A...-.....**

130 140 150 160 170

....|....|....|....|....|....|....|....|....|....|....|....

**Platy-1-1_NW_012163313.1:49684** **ACCT---AGG--------AAGCCC--TC--------ACCC--ATAT-------------**

**Platy-1-1_NW_012163535.1:17840** **.TG.---.TA--------C.TTTG--..--------.T..--..TAAAAA---------**

**Platy-1-1_NW_012162423.1:29766** **..ACATA-----------..C.T.--..--------.T..--..-.TAAAA--------**

**Platy-1-1_NW_012163535.1:32779** **..AC---..C--------....A.--..--------.T.T--.----------------**

**Platy-1-1_NW_012163536.1:46396** **..ACAGA.ACCTC---GA..C.T.--..--------.T..--....AAAAA--------**

**Platy-1-1_NW_012169090.1:29360** **..A.TA-.AA--------..C.T.--.A--------.T..--..TAAAA----------**

**Platy-1-1_NW_012170313.1:34330** **..ACAGA-----------..C.T.--..--------.T..--..-.AAAAA--------**

**Platy-1-1_NW_012170424.1:19233** **..A.---..A--------..CTT.--..--------.T.T-------------------**

**Platy-1-1_NW_012171090.1:58916** **..A.AGA-----------..C.G.--..--------.A..--.----------------**

**Platy-1-1_NW_012171312.1:20970** **..ACAGA-----------..C.T.--..--------.T..--..-.AAAAA--------**

**Platy-1-1_NW_012171312.1:22155** **G.A.---...--------....TT--..--------.T.T--.----------------**

**Platy-1-1_NW_012172090.1:24950** **..AGAGA-----------..C.T.--.T--------.T..--..-.AAAAA--------**

**Platy-1-1_NW_012173534.1:35510** **.TG.---.TA--------..C.T.--..--------.T..--..TA-------------**

**Platy-1-1_NW_012175091.1:28244** **..AC---..A--------....T.--..--------.T.T--.----------------**

**Platy-1-1_NW_012176869.1:33712** **..AC---..A--------..C.T.--..--------.T..--..---------------**

**Platy-1-1_NW_012177091.1:48611** **.TG.---.TA--------.GC.T.--..--------.T.T--..TAAAAA---------**

**Platy-1-1_NW_012177646.1:34834** **..T.---..C--------..C.T.--..--------GT..--..TACAAAA--------**

**Platy-1-1_NW_012177979.1:10635** **..GC---.TA--------..C.T.--..--------.T..--..TAAAA----------**

**Platy-1-1_NW_012178646.1:26805** **..G.---.TA--------..T.T.--..--------.T..--..TAA------------**

**Platy-1-1_NW_012183958.1:14675** **..ACAGA-----------..A.T.--..--------.T..--..TGAAA----------**

**Platy-1-1_NW_012184014.1:30269** **.TG.---.TA--------..C.T.--.G--------.T..--..TAA------------**

**Platy-1-1_NW_012184214.1:36295** **..A.AGA-----------..C.T.--..--------.T..--..-.AAAA---------**

**Platy-1-1_NW_012184536.1:15341** **..A.ATA-----------..CTTG--..--------.T..--..TAAAAAAA-------**

**Platy-1-1_NW_012184613.1:35624** **....---.TA--------..C.T.--..--------.T..--T..AAAAAAAA------**

**Platy-1-1_NW_012184646.1:76762** **..AGAGA.A-------------..--..TC------.T..--..TAAAAA---------**

**Platy-1-1_NW_012184647.1:17260** **..A.---...--------.....T--..--------.T.T--.----------------**

**Platy-1-1_NW_012184746.1:45925** **..A.AGG-----------..C.T.--C.--------.T..--.----------------**

**Platy-1-1_NW_012184979.1:12918** **G.A.GGA-----------..C.T.--..--------.T.T--..---------------**

**Platy-1-1_NW_012185580.1:14037** **..A.AGA-----------..C.T.--..--------.T..--..-.AAAAA--------**

**Platy-1-1_NW_012185646.1:80324** **..T.---..C--------..C.T.--..--------.T..--..GGAAA----------**

**Platy-1-1_NW_012186090.1:22025** **..A.---...--------..A...--..--------.T.T--..G.AACAAAG------**

**Platy-1-1_NW_012186313.1:66308** **..A.---...--------......--..--------.T.T--G----------------**

**Platy-1-1_NW_012187646.1:68445** **..A.---..C--------......--..--------.T.--------------------**

**Platy-1-1_NW_012187757.1:36641** **..AC---..A--------......--.T--------GT.T--...--------------**

**Platy-1-1_NW_012187946.1:15918** **..AC---..A--------..C...--..--------.T..--..T.TAAAA--------**

**Platy-1-1_NW_012188868.1:11813** **..G.GGA-----------..C.T.--..--------.T..--.----------------**

**Platy-1-1_NW_012189090.1:4979-** **..A.---..AT-------..C...--G.--------.T..--G.TAAAAA---------**

**Platy-1-1_NW_012189423.1:15745** **.TGC---.TA--------..C.T.--.G--------.T..--..TAAAA----------**

**Platy-1-1_NW_012189423.1:49188** **..A.---...--------......--.--------------------------------**

**Platy-1-1_NW_012189424.1:43236** **.TG.---.TA--------..C.T.--..--------.T..--..TA-------------**

**Platy-1-1_NW_012190534.1:27184** **..A.GTA-----------..C.T.--..--------.TT.--..T.AAAAAA-------**

**Platy-1-1_NW_012190979.1:22614** **..A.--TTA---------..C.T.--.T--------.TG.--..TAAAAA---------**

**Platy-1-2_NW_012164091.1:26806** **..A.-AAG----------..C.T.--..A--------TTT--..T.AAAA---------**

**Platy-1-2_NW_012164091.1:26906** **..A.-AAG----------..C.T.--..A--------TTT--..T.AAA----------**

**Platy-1-2_NW_012165424.1:13538** **..A.-AAG----------..C.T.--..A--------TT.--..T.AAAAAA-------**

**Platy-1-2_NW_012169090.1:16710** **....-AAG----------..C.T.--..A--------TT.--..T.AAAA---------**

**Platy-1-2_NW_012169535.1:14903** **..A.AGA-----------G...T.--..--------.T..--.CTGAAAAA--------**

**Platy-1-2_NW_012172423.1:21747** **..A.AGA-----------....G.--..--------.T..--..TGGAAA---------**

**Platy-1-2_NW_012172535.1:90777** **..A.-AAG----------C.C.T.--..AA-------TT.--..T.AAAAAA-------**

**Platy-1-2_NW_012173534.1:79384** **..A.-AAG----------..C.T.--..A--------.T.--..T.AAAAAA-------**

**Platy-1-2_NW_012176424.1:26496** **..A--TA---------AG..C.T.--..--------.TT.--.CT.AAAA---------**

**Platy-1-2_NW_012184158.1:19413** **..A.-AAG----------..C.TG--..A--------TT.--..T--------------**

**Platy-1-2_NW_012184202.1:16754** **.TA--TG.---------G..C.TG--..--------.TT.--..T.AA-----------**

**Platy-1-2_NW_012184980.1:57576** **..A--TAC---------AG.A.T.--..--------.TT.--..TGAA-----------**

**Platy-1-2_NW_012185302.1:16119** **..AGAGA-----------....T.--..--------CT..--G.TGAAA----------**

**Platy-1-2_NW_012187201.1:60133** **..A.AGA-----------..CAT.--..--------.T.T--..-.GAAA---------**

**Platy-1-2_NW_012187202.1:21081** **..A.-AG.----------..C.T.--..A--------TA.--..T.AAAA---------**

**Platy-1-2_NW_012187325.1:43653** **.TA.-AAG----------..C.TA--..A--------TT.--..T.AAAAAA-------**

**Platy-1-2_NW_012188646.1:21539** **..A.-AAG----------..C.T.--..A--------TT.--.CT.AAAAA--------**

**Platy-1-2_NW_012189423.1:10665** **G.A.AAA-----------..C...--..--------.T..--..TGA------------**

**Platy-1-2_NW_012189423.1:12584** **..AAAGA-----------C.C.T.--..A--------T.---..TGAAA----------**

**Platy-1-2_NW_012189424.1:90770** **..A.-AAG----------..C.T.--..A--------TT.--..TA-------------**

**Platy-1-2_NW_012189757.1:60581** **..A.-AAG----------..C.T----.T--------TT.--..T.GAAAA--------**

**Platy-1-2_NW_012190868.1:93211** **..A--TA.---------G..C.T.--..--------.TT.--..TGGAA----------**

**Platy-1-2a_NW_012162647.1:5295** **..A--TA---------AG..C.T.--..--------.TT.--G.T.AAA----------**

**Platy-1-2a_NW_012163869.1:6833** **..G.-AAG----------..C.T.--..A---------TT--.ATAAAAAA--------**

**Platy-1-2a_NW_012164202.1:3713** **..-A-TA.---------G..C.T.--..--------.TT.--..---------------**

**Platy-1-2a_NW_012164424.1:6158** **.TA.-AT.--AAAAAAAA.GC.T.--..A--------TT.--..TAAAAAA--------**

**Platy-1-2a_NW_012165424.1:1861** **..A--TA-------------CTTA--..--------.TT.--G.T.AAA----------**

**Platy-1-2a_NW_012167424.1:7138** **..A.-AA.----------..C.T.--..A--------TT.--..TAAAA----------**

**Platy-1-2a_NW_012167646.1:1017** **..A--TA.---------G.CC.T.--.A--------..T.--..T.-------------**

**Platy-1-2a_NW_012167868.1:8952** **.GA--TA---------AG..C.T.--C.--------.TTA--T.TAAC-----------**

**Platy-1-2a_NW_012170868.1:3906** **..A.-AA.----------.GC.T.--..A--------TT.--..TAAAAAAAAA-----**

**Platy-1-2a_NW_012171312.1:1107** **..A--TA---------AA..C.T.--..--------..T.--G.TAACA----------**

**Platy-1-2a_NW_012172535.1:1491** **..A.-TA.AAAAAAAAAA.CC.T.--..A--------TT.--..TAAAAAAAAA-----**

**Platy-1-2a_NW_012173534.1:4251** **..G--TA---------AG..C.T.--C.--------.TT.--..TAAAAA---------**

**Platy-1-2a_NW_012173535.1:9675** **..A.-AAG----------..C.T.--..A--------TT.--..T.AA-----------**

**Platy-1-2a_NW_012176424.1:1014** **..A--TA.---------G..C.T.--..--------.TT.--G.TAAAA----------**

**Platy-1-2a_NW_012176868.1:1095** **..G--TA.---------G..CTT.--..--------.TT.--..TAAAA----------**

**Platy-1-2a_NW_012177979.1:9460** **..A.-AT.---------A..C.T.--C.A--------TT.--..TAAAAAAC-------**

**Platy-1-2a_NW_012178424.1:8756** **..A.-GAG----------..C.TG--..A--------TT.--..TAAAA----------**

**Platy-1-2a_NW_012179535.1:1101** **...--CA---------AG..C------.--------.TT.--..TAAAA----------**

**Platy-1-2a_NW_012183869.1:1597** **..A.-AAG----------..C.T.--..A--------TT.--..TAAAA----------**

**Platy-1-2a_NW_012184025.1:1425** **..A--TA---------AG..C.T.--..--------.TT.--..TAAAA----------**

**Platy-1-2a_NW_012184091.1:5783** **..A--TA---------AG..C.T.--..--------.TTG--G.CAAAAA---------**

**Platy-1-2a_NW_012184202.1:1281** **..A--TAT--------AA..C.TT--C.--------.TT.--..TAAAA----------**

**Platy-1-2a_NW_012184358.1:8743** **..A--TA---------AG..C.T.--..--------..T.--..TAAAA----------**

**Platy-1-2a_NW_012184757.1:4571** **..A--TG.---------G..C.T.--..--------.TT.--...AAAATA--------**

**Platy-1-2a_NW_012184802.1:5276** **..A--TA---------AG..C.T.--..--------.TT.--..TAAAA----------**

**Platy-1-2a_NW_012184857.1:1202** **..G--TA---------AG..C.AA--..--------.TT.--..TAAAA----------**

**Platy-1-2a_NW_012184868.1:5356** **..A.-AAG----------..C.T.--..A--------TT.--..TAAAAAAA-------**

**Platy-1-2a_NW_012185025.1:8158** **..A--TA---------GA..A.T.--..--------.TT.--..TAAAAA---------**

**Platy-1-2a_NW_012185313.1:4572** **..A--TA---------AG..C.T.--..--------.TTA--..TAAAAA---------**

**Platy-1-2a_NW_012186868.1:3979** **.TA--TA---------AG..C.T.--..--------.TT.--..TAAAAAA--------**

**Platy-1-2a_NW_012187269.1:8608** **.TA--TA.---------G..C.T.--..--------.TT.--..TAA------------**

**Platy-1-2a_NW_012187424.1:6175** **..A.-AAG----------....T.--.TA--------TT.--..T.AAAAAAAA-----**

**Platy-1-2a_NW_012188312.1:2203** **..A--TA.---------G..C.T.--..--------.TTT--G.TAAAAA---------**

**Platy-1-2a_NW_012188646.1:2664** **.--.-TT.---------A...GTT--..--------.TTG--..T.AAAA---------**

**Platy-1-2a_NW_012189312.1:9461** **.TA.-AT.TAAAAAAAAA.GC.T.--AAA--------TT.--..T.AAAA---------**

**Platy-1-2a_NW_012189423.1:1252** **..-A-TA.------------CTT.--..--------.TT.--..T.GAAAA--------**

**Platy-1-2a_NW_012191312.1:2658** **..G.-AA.----------..C.T.--..A--------TT.--...AAAA----------**

**Platy-1-2b_NW_012162425.1:6193** **..A.---..C--------.....T--..--------.T.T--....AGAAA--------**

**Platy-1-2b_NW_012165757.1:1879** **..A.AGA-----------..C...--..--------TT..-------------------**

**Platy-1-2b_NW_012165757.1:2715** **G.A.AGA-----------G...T.--..--------.T.A--..TGAAAAAA-------**

**Platy-1-2b_NW_012165980.1:1672** **..A.GAA.AAACAATAAA.CAA..--..TC-------A..--..TAAAAA---------**

**Platy-1-2b_NW_012166091.1:5131** **..A.---..A--------..C.T.--..--------TT..--..TAAAA----------**

**Platy-1-2b_NW_012166313.1:3251** **..A.---.A.--------..A.T.--..--------.T..--..TAAAAT---------**

**Platy-1-2b_NW_012166535.1:5728** **..A.AGA-----------G.A.T.--..--------.T..--..T.AGAAA--------**

**Platy-1-2b_NW_012166868.1:4843** **..ACAGA-----------T...T.--..--------.T..--..TGAAAA---------**

**Platy-1-2b_NW_012167202.1:5072** **..A--TA.---------G.GCA..--.A--------.T.--------------------**

**Platy-1-2b_NW_012167313.1:2947** **G.ACAGA-----------..C.T.--..--------.T..--..-.AAAAA--------**

**Platy-1-2b_NW_012169313.1:3109** **.TA.AGA-----------..C.T.--..--------.T..--..TAAAA----------**

**Platy-1-2b_NW_012172201.1:9584** **..A.AAA-----------..C.T.--..--------.T..--..TAA------------**

**Platy-1-2b_NW_012173534.1:2304** **..A.AGA-----------..C.A.--..--------.T.T--..CAAAAA---------**

**Platy-1-2b_NW_012173534.1:2643** **..A.---...--------....T.--..--------.T.T--.----------------**

**Platy-1-2b_NW_012173535.1:2973** **..A.AGA-----------..C.T.--..--------....--.A.AAAA----------**

**Platy-1-2b_NW_012174646.1:7972** **..A.GAA-----------..C.T.--A.--------.T..--..T--------------**

**Platy-1-2b_NW_012175796.1:638-** **..A.---...--------....T.--..--------.T.T--..---------------**

**Platy-1-2b_NW_012180543.1:1009** **..A.TTA.AAAAAAAAAA..CA..--..TC------.T..--..TAAAA----------**

**Platy-1-2b_NW_012180868.1:6439** **..A.-AA.----------..T.T.--..A--------T..--..C--------------**

**Platy-1-2b_NW_012181646.1:7893** **..A.AGA-----------..C.T.--..--------.T..--G.TAAA-----------**

**Platy-1-2b_NW_012182423.1:7347** **..A.AGA-----------..C.TT--..--------.T..--.CT--------------**

**Platy-1-2b_NW_012183534.1:1116** **..A.AAA-----------.TC.T.--..--------.T..--.----------------**

**Platy-1-2b_NW_012183534.1:1895** **..ACAGA-----------....T.--..--------.T.T--..TGAAAAAA-------**

**Platy-1-2b_NW_012183868.1:7418** **..A.---..A--------..C.T.--..--------.T..--..TAAAAA---------**

**Platy-1-2b_NW_012183869.1:1188** **..A-CAA---------A------------------------------------------**

**Platy-1-2b_NW_012184424.1:3175** **.AA---------------..CTT.--..--------.T..--.C---------------**

**Platy-1-2b_NW_012185114.1:1027** **..A.TAA-----------..C.T.--.---------.T..--..TAAAAAA--------**

**Platy-1-2b_NW_012186090.1:1197** **..AAAAACAATTTGTTA------------------------------------------**

**Platy-1-2b_NW_012186202.1:4485** **..A.-TA.---------A..C.T.--..--------.T..--..T.AAA----------**

**Platy-1-2b_NW_012186313.1:7737** **..A.AAA-----------..CTT.--..--------.T..--..T--------------**

**Platy-1-2b_NW_012186424.1:3482** **..A.GGA-----------..C.T.--..--------CT..--..TAAAAA---------**

**Platy-1-2b_NW_012187424.1:5967** **..A.AGA-----------G...T.--..--------.T..--..TGGAA----------**

**Platy-1-2b_NW_012188090.1:1455** **..A.AGA-----------G...T.--..--------.T..--..TGGAAA---------**

**Platy-1-2b_NW_012188090.1:1622** **.TA.---..A--------.GC.T.--.T--------.T..--..T--------------**

**Platy-1-2b_NW_012188646.1:1667** **..A.AAA-----------..C...--..--------.T..--..T.TAAAAAAAG----**

**Platy-1-2b_NW_012188757.1:8687** **..A.-TA.----------..---------------------------------------**

**Platy-1-2b_NW_012189090.1:5283** **..A.AAA-----------..C.T.--..--------.T..--..TAA------------**

**Platy-1-2b_NW_012189423.1:1292** **..A--GA-----------G...T.--.A--------.T.A--..TGAA-----------**

**Platy-1-2b_NW_012189757.1:6380** **..A.AGA-----------..T.T.--A.--------.T..--..T.AAAAAA-------**

**Platy-1-2b_NW_012190534.1:2742** **..A.AGA-----------.GC.T.--..--------.T..--..T-AGAA---------**

**Platy-1-2b_NW_012190646.1:3121** **..A.AGA-----------G.C.T.--..--------.T..--..T.AAAA---------**

**Platy-1-2b_NW_012191312.1:2401** **..A.AGA-----------..C.T.--..--------.T..--..TAAA-----------**

**Platy-1-3_NW_012162423.1:14651** **..A.-AAG----------..C.T.--.TA--------TT.--..TAAAAAT--------**

**Platy-1-3_NW_012163091.1:14454** **..A.-AG.----------..C.T.--..A--------T..--.A.AAAA----------**

**Platy-1-3_NW_012163202.1:30198** **..A--TA---------AG..C.T.--..--------.TTA--..---------------**

**Platy-1-3_NW_012164869.1:60849** **..A.-AAT----------..C.T.--..A--------TT.--..TAAA-----------**

**Platy-1-3_NW_012165198.1:5928-** **..A.-AAG----------..C.TG--A.A--------TT.--..T.AAA----------**

**Platy-1-3_NW_012165535.1:14696** **..A--TA---------AG..C.T.--..--------.TT.--.CTAAAA----------**

**Platy-1-3_NW_012167535.1:56481** **..A.-AG.----------..C.T.--..A--------T.T--..T.AAA----------**

**Platy-1-3_NW_012170313.1:12544** **..A.-AAG----------..C.T.--C.A--------TT.--..T.AAAAA--------**

**Platy-1-3_NW_012170535.1:46745** **..A.-AAG----------..ATG.--..A--------TT.--..T.AAA----------**

**Platy-1-3_NW_012173534.1:14650** **..A.-AAG----------..C.T.--..A--------TT--------------------**

**Platy-1-3_NW_012173534.1:34177** **.TAA-TAG---------AG.A.T.--..TCT----C.TG.--..TGAAA----------**

**Platy-1-3_NW_012174202.1:58320** **..A.AGA-----------...-TT--.T--------.T..--..TAA------------**

**Platy-1-3_NW_012174424.1:28994** **..A--TA---------AG..C.T.--..--------..T.--.CTAAAAA---------**

**Platy-1-3_NW_012178646.1:42536** **..A.-AAG----------T.C.T.--..A--------TT.--..TAAAAAT--------**

**Platy-1-3_NW_012179646.1:13674** **..A.-AA.----------..CAT.--.TA--------TT.--..TAAC-----------**

**Platy-1-3_NW_012179646.1:13765** **..A.-AA.----------..CAT.--.TA--------TT.--..TAACC----------**

**Platy-1-3_NW_012180757.1:22137** **..A.-GA.----------..C.T.--A.A--------T..--..TAACAAA--------**

**Platy-1-3_NW_012181868.1:10555** **..A--TA.---------G..A.T.--..--------.TT.--..TAAA-----------**

**Platy-1-3_NW_012183892.1:21329** **.TA--TA---------AG..C.TG--..--------.TT.--..TAAAAAA--------**

**Platy-1-3_NW_012184003.1:21311** **....-AAG----------G.CAA.--..A--------------.T.A------------**

**Platy-1-3_NW_012184092.1:29931** **..A--TA---------AG..CTT.--..--------.TT.--.CTAGAA----------**

**Platy-1-3_NW_012184092.1:47861** **..ACAGA-----------C.C.T.--..--------.T.T--.CT.AAAAAA-------**

**Platy-1-3_NW_012185313.1:81785** **..A.AGA-----------....T.--..--------TT..--.CTA-------------**

**Platy-1-3_NW_012185535.1:12734** **..A--TA---------AG..C.T.--..--------.TT.--..TAAAAA---------**

**Platy-1-3_NW_012185757.1:66791** **..-A-TA.---------GT.C.T.--..--------GTT.--..T.AAA----------**

**Platy-1-3_NW_012186720.1:3413-** **..A.-AAG----------T.C.T.--..A--------TT.--..TAAAA----------**

**Platy-1-3_NW_012186757.1:60120** **..A.---..A--------..C.T.--..--------.TT.--..TGCAA----------**

**Platy-1-3_NW_012188090.1:38232** **..T.-AAG----------..CGT.--..A--------TTG--..T.AAAAA--------**

**Platy-1-3_NW_012188458.1:41468** **..A.-GAG----------..C.T.--..A--------TT.--..TAAA-----------**

**Platy-1-3_NW_012191423.1:67979** **..A.-AAG----------..C.T.--.TA--------TT.--..T.AAA----------**

**Platy-1-4_NW_012173980.1:20137** **..G--TA.---------G..CAT.--..--------.TT.--..TAAAAAAAAA-----**

**Platy-1-4_NW_012189423.1:11596** **.TA--TG.---------G..C.T.--..--------.TT.--..TAAAAA---------**

**Platy-1-4_NW_012189868.1:48523** **.TA--TG.---------G..C.T.--..--------.TT.--..TA-------------**

**Platy-1-4_NW_012190201.1:56706** **..A.-AAG----------..CTT.--..A--------TT.--..T.AAAA---------**

**Platy-1-4a_NW_012162647.1:5343** **.TG--TG.---------G..C.T.--..--------.TT.--..TGAA-----------**

**Platy-1-4a_NW_012162869.1:5364** **.TA--TG.---------G..C.T.--..--------.TT.--.CTGAAAA---------**

**Platy-1-4a_NW_012162869.1:5365** **.TA--TG.---------G..C.T.--..--------.TT.--.CTGAAAA---------**

**Platy-1-4a_NW_012163158.1:1929** **.TA--TG.---------G..C.T.--..--------.TT.--..TAAAA----------**

**Platy-1-4a_NW_012163535.1:2382** **.TA--TG.---------G..C.T.--..--------.TT.--..T.AA-----------**

**Platy-1-4a_NW_012163980.1:1247** **.TA--TG.---------G.GC.T.--..--------.TT.--..TAAAAAA--------**

**Platy-1-4a_NW_012164202.1:2548** **..A--TG.---------G..C.T.--..--------.TT.--..T.AAAA---------**

**Platy-1-4a_NW_012164424.1:2374** **.TA--TG.---------G..C.T.--..--------.TT.--..TAAAA----------**

**Platy-1-4a_NW_012164646.1:1456** **..A--TA.---------G..C.T.--..--------..T.--..TAAAAAAA-------**

**Platy-1-4a_NW_012165091.1:1698** **.TA--TG.---------G..C.T-------------.TTA--.A.--------------**

**Platy-1-4a_NW_012165091.1:2771** **.TA--TG.---------G..C.T.--..--------GTT.--..TAAA-----------**

**Platy-1-4a_NW_012165091.1:5564** **..G--TG.---------G..A.T.--..--------.TT.--..TG-------------**

**Platy-1-4a_NW_012165535.1:1152** **.TA--TG.---------G..C.T.--..--------.TT.--..TAAAA----------**

**Platy-1-4a_NW_012165757.1:4481** **.TA--TG.---------G..C.T.--..--------.T-.--..TAAAA----------**

**Platy-1-4a_NW_012165757.1:4482** **.TA--TG.---------G..C.T.--..--------.T-.--..TAAAA----------**

**Platy-1-4a_NW_012165757.1:8542** **.TA--TG.---------G..C.T.--..--------.TT.--..TAAAAAAAA------**

**Platy-1-4a_NW_012165980.1:3779** **.TA--TG.---------G..C.T.--..--------.TT.--..TAAAA----------**

**Platy-1-4a_NW_012166202.1:2027** **.TA--TG.---------G..C.T.--..--------.AT.--G.T.AAAA---------**

**Platy-1-4a_NW_012166646.1:3729** **.TA--TG.---------G..C.T.--..--------.TT.--.CTAAAA----------**

**Platy-1-4a_NW_012167202.1:4991** **.TA--TG.---------G..C.T.--..--------.TT.--..T.AAAA---------**

**Platy-1-4a_NW_012167313.1:3173** **.TA--TG.---------G..C.T.--..--------.TT.--..TAAAA----------**

**Platy-1-4a_NW_012167424.1:2226** **.TA--TG.---------G.CC.T.--..--------.TT.--...AAAAA---------**

**Platy-1-4a_NW_012167868.1:6778** **.TA--TG.---------G..C.T.--..--------.TT.--..T.AAAA---------**

**Platy-1-4a_NW_012168313.1:1440** **TT---------------G..C.T.--..--------.TT.--..TAAAAAT--------**

**Platy-1-4a_NW_012168424.1:4326** **.TA--TG.---------G..C.T.--..--------.TT.--..TAAAAAA--------**

**Platy-1-4a_NW_012168646.1:1504** **.TA--TG.---------G..T.T.--..--------.TT.--..T.AAAA---------**

**Platy-1-4a_NW_012168979.1:8062** **.TA--TG.---------G..C.T.--..--------.TT.--..T.AAAA---------**

**Platy-1-4a_NW_012169090.1:2106** **.TA--TG.---------G..C.T.--..--------.TTG--..---------------**

**Platy-1-4a_NW_012169090.1:4300** **..A--TA.---------G..A.T.--..--------.TT.--..TAAAA----------**

**Platy-1-4a_NW_012169091.1:2859** **..A--TG.---------G..A.TG--..--------.TT.--..TGAAA----------**

**Platy-1-4a_NW_012169313.1:2654** **.TA--TG.---------G..C.T.--..--------.TT.--..TAAAAA---------**

**Platy-1-4a_NW_012169535.1:7280** **.TA--TG.---------G..C.T.--..--------.TT.--.----------------**

**Platy-1-4a_NW_012169979.1:4186** **..A--TA.--------AG..C.T.--..--------.TT.--..TA-------------**

**Platy-1-4a_NW_012170201.1:1053** **..A.-AAG----------..CTT.--..A--------TT.--..TAAAAA---------**

**Platy-1-4a_NW_012171126.1:2521** **.TA--TG.---------G..C.T.--..--------.TT.--..TAATA----------**

**Platy-1-4a_NW_012171201.1:1320** **.TA--TG.---------G..C.T.--..--------.TT.--..TAAA-----------**

**Platy-1-4a_NW_012171312.1:1877** **.TA--TG.---------G..C.T.--..--------.TT.--..TAAAA----------**

**Platy-1-4a_NW_012171312.1:5539** **.TA--TG.---------G..C.T.--..--------.TT.--..TAA------------**

**Platy-1-4a_NW_012173201.1:4719** **..A--TG.---------G..C.T.--..--------.TT.--..TAAA-----------**

**Platy-1-4a_NW_012173312.1:2571** **..A--TG.---------G..C.T.--..--------.TT.--..TA-------------**

**Platy-1-4a_NW_012173758.1:3202** **.TA--TG.---------G..C.T.--..--------.TT.--..TAAA-----------**

**Platy-1-4a_NW_012173758.1:3282** **.TA--CG.---------G..C.T.--..--------.TT.--..TAAAAAAA-------**

**Platy-1-4a_NW_012174424.1:7477** **.TA--TG.---------G.GC.T.--..--------.TT.--..TAAAAAAA-------**

**Platy-1-4a_NW_012174646.1:1101** **.TA--TG.---------G..C.T.--..--------.TT.--..T.AAAAA--------**

**Platy-1-4a_NW_012175646.1:4412** **.TA--TG.---------G..C.T.--..--------.TT.--..T.AAA----------**

**Platy-1-4a_NW_012176202.1:1353** **.TA--TG.---------G..T.T.--..--------.TTT--..TAAAAA---------**

**Platy-1-4a_NW_012176313.1:3041** **.TA--TG.---------G..CTT.--..--------.TT.--..TAAAAA---------**

**Platy-1-4a_NW_012176535.1:1424** **..A--TG.---------G..C.T.--..--------.TTA--..TGAAAA---------**

**Platy-1-4a_NW_012176646.1:1884** **..A--TA.---------G..C.T.--..--------.TT.--..TAAAAAA--------**

**Platy-1-4a_NW_012176757.1:2674** **.TA--TG.---------G..C.T.--..--------.TT.--..TAA------------**

**Platy-1-4a_NW_012177979.1:2731** **..A--TA.---------G..C...--..--------.TT.--..TCAAAAAA-------**

**Platy-1-4a_NW_012178025.1:1167** **.TA--TG.---------G..C.T.--..--------.TT.--..T.AAAA---------**

**Platy-1-4a_NW_012178091.1:1955** **.TA--TG.---------G..C.T.--..--------.TT.--..TAAAAA---------**

**Platy-1-4a_NW_012178535.1:1006** **.TA--TG.---------G..C.T.--..--------.TT.--..TAAAAA---------**

**Platy-1-4a_NW_012178757.1:4212** **.TA--TG.---------G..C.T.--..--------.TT.--..T.AA-----------**

**Platy-1-4a_NW_012181312.1:9214** **.TA--TG.---------G..C.T.--..--------.TT.--..TAAAGTT--------**

**Platy-1-4a_NW_012182535.1:1154** **.TA--TG.---------G..C.T.--..--------.TT.--..T.AAA----------**

**Platy-1-4a_NW_012183201.1:3224** **.TA--TG.---------G..C.T.--..--------.TT.--..T.AAAA---------**

**Platy-1-4a_NW_012183802.1:7811** **.TA--TG.---------G..C.T.--..--------.TT.--..TAAA-----------**

**Platy-1-4a_NW_012183813.1:5526** **.TA--TG.---------G..C.T.TC----------.TT.--.A.AAAAA---------**

**Platy-1-4a_NW_012183824.1:1101** **.TA--TA.---------G..C.T.--..--------.TT.--.CTGGAAAA--------**

**Platy-1-4a_NW_012183869.1:1041** **.TA--TG.---------G..C.T.--..TC------.TT.--..TAAAA----------**

**Platy-1-4a_NW_012183869.1:1360** **.TA--TG.---------G..C.T.--..--------.TT.--..TAAAAA---------**

**Platy-1-4a_NW_012183869.1:5673** **.TA--TG.---------G..C.T.--..--------.TT.--..T--------------**

**Platy-1-4a_NW_012183881.1:1201** **..A--TA.---------G..C.T.--..--------.TT.--..TAAAA----------**

**Platy-1-4a_NW_012183958.1:1156** **.TA--TG.---------G..C.T.--..--------.TT.--..TAAAAA---------**

**Platy-1-4a_NW_012183980.1:1098** **..A.-A-------------.C.AT--..A--------TT.--..T.AAA----------**

**Platy-1-4a_NW_012184047.1:9856** **..A--TA.---------G..C.T.--..--------.TT.--..TAAAA----------**

**Platy-1-4a_NW_012184147.1:3563** **.TA--TG.---------G..C.T.--C.--------.TT.--..TAAAAAA--------**

**Platy-1-4a_NW_012184269.1:1219** **.TA--TG.---------G..C.T.--..--------.TT.--..TAAAA----------**

**Platy-1-4a_NW_012184480.1:8638** **.TA--TG.---------T..C.T.--..--------.TT.--..T.AAA----------**

**Platy-1-4a_NW_012184535.1:3014** **..A--TA.---------G..C.TG--..--------.TT.--..TAAAAA---------**

**Platy-1-4a_NW_012184535.1:7443** **.TA--TG.---------G..C.T.--..--------.TT.--..TAAAAA---------**

**Platy-1-4a_NW_012184602.1:1032** **.TA--TG.---------G..CTT.--..--------.TT.--..TGGAA----------**

**Platy-1-4a_NW_012184702.1:2422** **.TA--TG.---------G..C.T.--..--------.GT.--..TAAAA----------**

**Platy-1-4a_NW_012184735.1:1484** **.TA--TG.---------G..C.T.--..--------.TT.--..TAAAAA---------**

**Platy-1-4a_NW_012184902.1:5722** **.TA--TG.T------GAG..CTT.--..--------.TT.--..TA-------------**

**Platy-1-4a_NW_012184980.1:7322** **..A--TA.---------G..C.T.--..--------.TT.--..TAAA-----------**

**Platy-1-4a_NW_012185202.1:8626** **..A--CG.---------G..C.T.--..--------.TT.--..T.AAAAA--------**

**Platy-1-4a_NW_012185247.1:6115** **..A--TA.---------G..C.T.--..--------.TT.--..TAAAA----------**

**Platy-1-4a_NW_012185313.1:1077** **.TA--TG.---------G..C.T.--..--------.TT.--..TAAAAAA--------**

**Platy-1-4a_NW_012185313.1:9239** **.TA--TG.T------G--..C.T.--..--------.TT.--..TAAAG----------**

**Platy-1-4a_NW_012185424.1:2354** **..A--TG.---------G..C.T.--..--------.TT.--..TAAAAAAA-------**

**Platy-1-4a_NW_012185425.1:7570** **.TA--TG.---------G..C.T.--..--------.TT.--.ATAAAGAAA-------**

**Platy-1-4a_NW_012185458.1:2034** **.TA--TG.---------G..C.T.--..--------.TT.--..TAAAAA---------**

**Platy-1-4a_NW_012185757.1:1154** **.TA--TG.---------GT.C-T.--..--------.TT.--..TAAAAAATA------**

**Platy-1-4a_NW_012185757.1:1242** **.TA--TG.---------G..C.T.--..--------.TT.--..TAAAG----------**

**Platy-1-4a_NW_012185913.1:1882** **.TA--TG.---------G..C.T.--..--------.TT.--..TAAAAA---------**

**Platy-1-4a_NW_012185957.1:9479** **.TA--TG.---------G..C.T.--..--------.TT.--..TAAA-----------**

**Platy-1-4a_NW_012186091.1:6351** **.TA--TG.---------G..C.T.--..--------.TT.--..T.AAA----------**

**Platy-1-4a_NW_012186114.1:2462** **.TA--TG.---------G..C.T.--..--------.TT.--..T.AAA----------**

**Platy-1-4a_NW_012186313.1:4701** **.TA--TG.---------G..C.T.--..--------.TT.--..T.AAAA---------**

**Platy-1-4a_NW_012186535.1:5683** **.TA--TG.---------G..C.T.--.--------------------------------**

**Platy-1-4a_NW_012186646.1:6551** **.TA--TG.---------G..C.T.--..--------.TT.--..T.AAA----------**

**Platy-1-4a_NW_012186757.1:1166** **.TA--TG.---------G..C.TT--..--------.TT.--..TAAAA----------**

**Platy-1-4a_NW_012186757.1:1875** **.TA--CG.---------G..C.T.--..--------.TT.--..TA-------------**

**Platy-1-4a_NW_012186979.1:9295** **.TA--TG.---------G..C.T.--..--------.TT.--..---------------**

**Platy-1-4a_NW_012187202.1:5070** **..A--TG.---------G..A.T.------------.TT.--..TAAAAAAA-------**

**Platy-1-4a_NW_012187757.1:6197** **.TG--TG.---------G..C.T.--..--------.TT.--..TAA------------**

**Platy-1-4a_NW_012188090.1:6532** **.TA--TG.---------G..C.T.--..--------.TT.--..---------------**

**Platy-1-4a_NW_012188868.1:4269** **.TA--TG.---------G..C.T.--..--------.TT.--..TAAAAA---------**

**Platy-1-4a_NW_012189201.1:1245** **.TA--TG.---------G..C.T.--G.--------.TT.--..GGAAAAA--------**

**Platy-1-4a_NW_012189201.1:2745** **.TA--TG.---------G..C.T.--..--------.TT.--..T.TAAAAA-------**

**Platy-1-4a_NW_012189423.1:1301** **..A--TG.---------G.CC.T.--..--------.TT.--..TAAAA----------**

**Platy-1-4a_NW_012189423.1:2606** **.TA--TG.---------G..C.T.--..--------.TT.--..TAAAA----------**

**Platy-1-4a_NW_012189424.1:1588** **.TA--TG.---------G..C.T.--..--------.TT.--..TAAAA----------**

**Platy-1-4a_NW_012189979.1:1974** **.TA--TG.---------G.CC.T.--..--------.TT.--..T.TAAAAA-------**

**Platy-1-4a_NW_012189979.1:3723** **.TA--TG.---------G..C.T.--..--------.TT.--..TA-------------**

**Platy-1-4a_NW_012190534.1:1765** **.TA--TG.---------G..C.T.--..--------.TT.--..T.TAAAAA-------**

**Platy-1-4a_NW_012190757.1:6050** **..A--TA.---------G..C.T.--..--------.TT.--..TAAAA----------**

**Platy-1-4a_NW_012190868.1:3122** **.TA--TGG---------G..C.T.--..--------.TTT--..TAAAAA---------**

**Platy-1-4a_NW_012191090.1:1473** **.TA--TG.---------G..C.T.--..--------.TT.--..TAAAAA---------**

**Platy-1-4a_NW_012191090.1:7220** **.TA--TG.---------G..C.T.--..--------.TT.--..T.AAAAAAAA-----**

**Platy-1-4a_NW_012191201.1:4096** **.TA--TG.---------G..C.T.--..--------.TT.--..T.AAAAAA-------**

**Platy-1-4a_NW_012191423.1:5463** **.TA--TG.---------G..C.T.--..--------.TT.--..TAGA-----------**

**Platy-1-4b_NW_012162758.1:2572** **.TA--TG.T--------G..C.T.--..--------.TT.--..T.AAA----------**

**Platy-1-4b_NW_012162869.1:6381** **.TA--TG.---------G..C.T.--..--------.TT.--..TAAAA----------**

**Platy-1-4b_NW_012163202.1:1994** **.T---TG.---------G.GC.T.--..--------.TT.--..GAAAA----------**

**Platy-1-4b_NW_012163535.1:2530** **.TA--TG.---------G..C.T.--..--------.TT.--..TAA------------**

**Platy-1-4b_NW_012163535.1:2531** **.TA--TG.---------G..C.T.--..--------.TT.--..TAA------------**

**Platy-1-4b_NW_012163535.1:8297** **.TA--TG.---------G..C.T.--..--------.TT.--..TAAAA----------**

**Platy-1-4b_NW_012163647.1:6366** **.TA--TG.---------G..C.T.--..--------.TT.--..TAAAAA---------**

**Platy-1-4b_NW_012163926.1:5965** **.TA--TG.---------G..A.T.--..--------.TT.--..TAAAA----------**

**Platy-1-4b_NW_012164646.1:9630** **.TA--TG.---------G..C.T.--..--------.TT.--..TAAAAA---------**

**Platy-1-4b_NW_012165757.1:5703** **.TA--TG.---------G..C.T.--..--------.TT.--..TG-------------**

**Platy-1-4b_NW_012166868.1:1567** **.TA--TG.---------G..C.T.--..--------.TT.--..TGAAA----------**

**Platy-1-4b_NW_012167979.1:1849** **.TA--TG.---------G..C.T.--..--------.TT.--..---------------**

**Platy-1-4b_NW_012168979.1:4354** **.TA--TG.---------G..CTT.--..--------.TT.--..TGAAAA---------**

**Platy-1-4b_NW_012169202.1:2313** **.TA--TG.---------G..C.T.--..--------.TT.--..TAAAA----------**

**Platy-1-4b_NW_012169202.1:2314** **.TA--TG.---------G..C.T.--..--------.TT.--..TAAAA----------**

**Platy-1-4b_NW_012169313.1:3048** **.TA--TG.---------G..C.T.--..--------.TA.--..TAAAA----------**

**Platy-1-4b_NW_012172423.1:1435** **.TA--TG.---------G..C.T.--..--------.TT.--..TAAAAA---------**

**Platy-1-4b_NW_012172846.1:88-1** **.TA--TG.---------G..C.T.--..--------.TT.--..TAAAAA---------**

**Platy-1-4b_NW_012173423.1:6003** **.TA--TG.T--------G..C.T.--.A--------.TT.--..TAAAAA---------**

**Platy-1-4b_NW_012173534.1:2605** **.TA--TG.---------G..CAT.--..--------.TT.--..TAAAAA---------**

**Platy-1-4b_NW_012173536.1:2838** **.TA--TG.---------G..C.T.--..--------.TT.--..T.AAA----------**

**Platy-1-4b_NW_012174202.1:6446** **.TA--TG.---------G..C.T.--..--------.TA.--..TAAAAA---------**

**Platy-1-4b_NW_012175568.1:1042** **.TA--TG.---------G..C.T.--..--------.TA.--..TAA------------**

**Platy-1-4b_NW_012179090.1:4388** **.TA--TG.---------G..C.T.--..--------.TT.--..TAAA-----------**

**Platy-1-4b_NW_012179424.1:2256** **.TA--TG.---------G..C.T.--..--------.TT.--.CTAAAAAA--------**

**Platy-1-4b_NW_012180424.1:1829** **.TA--TG.---------G..C.T.--..--------.TT.--..TAAA-----------**

**Platy-1-4b_NW_012181312.1:4593** **.TA--TG.---------G..C.T.--..--------.TT.--..T.AAA----------**

**Platy-1-4b_NW_012181868.1:3187** **.TA--TG.---------G.CC.T.--..--------.TTA--.A.AAAAA---------**

**Platy-1-4b_NW_012182423.1:1093** **.TA--TG.---------G..C.T.--..--------.TT.--..TGAAAAA--------**

**Platy-1-4b_NW_012182979.1:1332** **.TA--TG.---------G..C.T.--..--------.TA.--..TAAAAA---------**

**Platy-1-4b_NW_012184003.1:1762** **..----G.---------G..C.T.--..--------.TT.--..TGAA-----------**

**Platy-1-4b_NW_012184313.1:9985** **.TA--TG.---------G..C.T.--..--------.TT.--..T.AAAA---------**

**Platy-1-4b_NW_012184436.1:2031** **.TA--TG.---------G..C.T.--..--------.TT.--..TGAAAA---------**

**Platy-1-4b_NW_012184513.1:8005** **.TA--TG.---------G..C.T.--..--------.TA.--..TAAAAA---------**

**Platy-1-4b_NW_012184646.1:1081** **.TA--TG.---------G..C.T.--..--------.TT.--..TAAA-----------**

**Platy-1-4b_NW_012184868.1:5660** **.TA--TG.---------G..C.T.--..--------.TT.--..TAAAA----------**

**Platy-1-4b_NW_012185091.1:4580** **.TA--TG.---------G..C.T.--..--------.TT.--..TGAAAA---------**

**Platy-1-4b_NW_012185202.1:3769** **.TA--TG.---------G..C.T.--..--------.TA.--.----------------**

**Platy-1-4b_NW_012185424.1:3710** **.TA--TG.---------G..C.T.--..--------.TT.--...AAAATAA-------**

**Platy-1-4b_NW_012185424.1:3916** **.TA--TG.---------G..C.T.--..--------.TT.--..TAAAAA---------**

**Platy-1-4b_NW_012185646.1:1492** **.TA--TG.---------G..C.T.--..--------.TT.--..TGGA-----------**

**Platy-1-4b_NW_012185757.1:1106** **.TA--TG.---------G..C.T.--..--------.TT.--..T--------------**

**Platy-1-4b_NW_012185868.1:3666** **.T---TG.---------G.CC.T.--..--------.TT.--..TAAAA----------**

**Platy-1-4b_NW_012186091.1:1130** **.TA--TG.---------G..C.T.--..--------.TT.--.CTAAAAA---------**

**Platy-1-4b_NW_012186202.1:1115** **.TA--TG.---------G..C.T.--..--------.TAT--..T.AAA----------**

**Platy-1-4b_NW_012186535.1:9707** **.TA--TG.---------G..A.T.--..--------.TT.--..CA-------------**

**Platy-1-4b_NW_012186979.1:1006** **.TA--TG.---------G..C.T.--..--------.TT.--..T--------------**

**Platy-1-4b_NW_012187313.1:1001** **.TA--TG.---------G..C.T.--..--------.TT.--..TAAAA----------**

**Platy-1-4b_NW_012188312.1:6490** **.TA--TG.---------G..C.T.--..--------.TT.--..TGAAA----------**

**Platy-1-4b_NW_012188480.1:1196** **.TA--TG.---------G..T.T.--..----TCTCGTT.--G.CAA------------**

**Platy-1-4b_NW_012189423.1:1089** **.TA--TG.---------G..C.T.--..--------.TA.--..TAA------------**

**Platy-1-4b_NW_012189423.1:1825** **.TA--TG.---------G..C.T.--..--------.TT.--..TAAT-----------**

**Platy-1-4b_NW_012189423.1:1887** **.TA--CG.---------G..C.T.--..--------.TT.--..TGAAAA---------**

**Platy-1-4b_NW_012190080.1:706-** **.TA--TG.---------G..C.T.--..--------.TT.--..TAAAAA---------**

**Platy-1-4b_NW_012190534.1:9595** **.TA--TG.---------G..C.T.--..--------.TT.--..TGA------------**

**Platy-1-4b_NW_012190868.1:1499** **.TA--TG.---------G..C.T.--..--------.TT.--..TGAAAA---------**

**Platy-1-4b_NW_012191201.1:6367** **.TA--TG.---------G..C.T.--..--------.TT.--..T.AAA----------**

**Platy-1-4b_NW_012191312.1:2418** **.TA--TG.---------G..C.T.--..--------.TT.--.CTGAAAAAAA------**

**Platy-1-4b3_NW_012172535.1:174** **.TA--TG.---------G..C.T.--..--------.TT.--..TGAAA----------**

**Platy-1-4b3_NW_012180979.1:316** **.TA--TG.---------G..C.T.--..--------.TT.--..TGAAAAA--------**

**Platy-1-4b3_NW_012181312.1:304** **.TA--TG.---------G..C.T.--..--------.TT.--..TGAAAA---------**

**Platy-1-4b3_NW_012184424.1:305** **.TA--TG.---------G..C.T.--..--------.TT.--..TGAAA----------**

**Platy-1-4b3_NW_012184591.1:794** **.TA--TG.---------G..T.T.--..--------.TT.--..TN-------------**

**Platy-1-4b3_NW_012184868.1:557** **.TA--TG.---------G..CAT.--..--------.TT.--..TGAAA----------**

**Platy-1-4b3_NW_012185158.1:100** **.TA--TG.---------G..C.T.--..--------.TT.--..TGAAA----------**

**Platy-1-4b3_NW_012185202.1:276** **.TA--TG.---------G..C.T.--..--------.TT.--..TGA------------**

**Platy-1-4b3_NW_012187201.1:328** **.TA--TG.---------G..C.T.--..--------.TT.--...GAAA----------**

**Platy-1-4b3_NW_012190535.1:324** **.TA--TG.T--------G..C.T.--..--------.TT.--..TAAAAAAA-------**

**Platy-1-5_NW_012163980.1:74925** **..-A-TA.---------G..C.T.--..--------.TT.--..TAA------------**

**Platy-1-5_NW_012164424.1:51747** **..A--TA---------AG..T.T.--..--------.TT.--..TAAA-----------**

**Platy-1-5_NW_012166868.1:18448** **..A--TA---------AG..C.T.--..--------.TT.--..TAAAA----------**

**Platy-1-5_NW_012167202.1:13968** **..A--TA---------AG..C.T.--..--------.TT.--..T.AAAA---------**

**Platy-1-5_NW_012169090.1:46811** **..A--TA---------AG..C.G.--..--------.TT.--..T.AAAA---------**

**Platy-1-5_NW_012173423.1:20889** **..G--TA---------A---C.T.--..--------.TT.--..TAGAA----------**

**Platy-1-5_NW_012178979.1:21624** **..A--TA---------AG..C.T.--..--------.TT.--..TAAAAAATA------**

**Platy-1-5_NW_012183869.1:10206** **..A--TA---------AG..C.T.--..--------.TT.--..TAAAAA---------**

**Platy-1-5_NW_012184203.1:40477** **..A--TA---------AG..C.T.--..--------.TT.--.CTAAAAA---------**

**Platy-1-5_NW_012184979.1:15607** **..A--TA---------AG..C.T.--..--------.TT.--..TAAA-----------**

**Platy-1-5_NW_012185158.1:92283** **G.A--TA---------AG.GC.T.--..--------.TT.--..TAAAA----------**

**Platy-1-5_NW_012185880.1:91983** **..A--TA---------AG..C.T.--..--------.TT.--..T.AAAA---------**

**Platy-1-5_NW_012185945.1:596-6** **.TA--TA.---------G..T.TT--..--------..T.--..TAAAAA---------**

**Platy-1-5_NW_012187313.1:37890** **..A--TA.---------G..C.T.--..--------.TT.--..TAAA-----------**

**Platy-1-5_NW_012166980.1:44419** **..A--TA---------AGC.C.T.--.T--------.TT.--.A.AA------------**

**Platy-1-5_NW_012167274.1:17-11** **..A--TA---------AGC.C.T.--.T--------.TT.--..TAAAA----------**

**Platy-1-5_NW_012169424.1:10725** **..A--TA---------AG..C.T.--..--------.TT.--...AA------------**

**Platy-1-5_NW_012178219.1:204-3** **...C-AAG----------..C.AA--..A--------TT.--..T.AAA----------**

Supplemental Figure 1: Alignment of all full-length Platy-1 elements ascertained from the owl monkey genome.

**A**

Supplementary Figure 2. Platy-1 element subfamily distribution based on PCR. The data used to construct this figure is based on a subset of 204 Platy-1 elements reported in Konkel et al 2016. A) Platy-1 element distribution based on subfamily and lineage-specificity. Lineage specific insertions are defined as NWM: the element is homozygous present in all New World Monkeys analyzed (see methods in Konkel et al. 2016 for the DNA panel used); Cebidae: the element is shared in marmosets, tamarin, cebus, squirrel and owl monkey, while absent from other NWM; Call: the element is shared in Callithrichinae (marmosets and tamarins); Marm: the element is shared in pygmy and common marmoset while absent from other NWM; cM: the element is lineage specific to common marmoset. B) Percent divergence distribution among and within the Platy-1 subfamilies. The lowest percent divergence is shown in red (0.0) and the highest percent divergence is shown in dark grey (20.0)

**B**
